# COVID-19 in Italy: Dataset of the Italian Civil Protection Department

**DOI:** 10.1016/j.dib.2020.105526

**Published:** 2020-04-10

**Authors:** Italian Civil Protection Department, Micaela Morettini, Agnese Sbrollini, Ilaria Marcantoni, Laura Burattini

**Affiliations:** a Italian Presidency of the Councils of Ministers, Roma, Italy; b Department of Information Engineering, Università Politecnica delle Marche, Ancona, Italy

**Keywords:** SARS-CoV-2, Coronavirus disease (COVID-19), Epidemiology, Virology, Infectious disease, Health emergency, Public health, Health policy

## Abstract

The database here described contains data of integrated surveillance for the “Coronavirus disease 2019” (abbreviated as COVID-19 by the World Health Organization) in Italy, caused by the novel coronavirus SARS-CoV-2. The database, included in a main folder called COVID-19, has been designed and created by the Italian Civil Protection Department, which currently manages it. The database consists of six folders called ‘aree’ (containing charts of geographical areas interested by containment measures), ‘dati-andamento-nazionale’ (containing data relating to the national trend of SARS-CoV-2 spread), ‘dati-json’ (containing data that summarize the national, provincial and regional trends of SARS-CoV-2 spread), ‘dati-province’ (containing data relating to the provincial trend of SARS-CoV-2 spread), ‘dati-regioni’ (containing data relating to the regional trend of SARS-CoV-2 spread) and ‘schede-riepilogative’ (containing summary sheets relating to the provincial and regional trends of SARS-CoV-2 spread). The Italian Civil Protection Department daily receives data by the Italian Ministry of Health, analyzes them and updates the database. Thus, the database is subject to daily updates and integrations. The database is freely accessible (CC-BY-4.0 license) at https://github.com/pcm-dpc/COVID-19. This database is useful to provide insight on the spread mechanism of SARS-CoV-2, to support organizations in the evaluation of the efficiency of current prevention and control measures, and to support governments in the future prevention decisions.

Specifications table

**Table:** 

Subject	Public Health and Health Policy
Specific subject area	Infectious diseases and virology
Type of data	Table, Chart
How data were acquired	Official records of national and regional healthcare system
Data format	Raw and analyzed
Parameters for data collection	Data of integrated surveillance for the COVID-19 in Italy, caused by the novel SARS-CoV-2. In particular, the database includes daily epidemiological data (since Feb 24^th^, 2020) at national, regional and provincial level. Indication of geographical areas interested by containment measures completes the database.
Description of data collection	Epidemiological data about SARS-CoV-2 are daily collected by the regional institutions that send them to the Italian Ministry of Health. The Italian Ministry of Health, in turn, sends the data to the Italian Civil Protection Department.
Data source location	Italian Civil Protection Department, Rome, Italy
Data accessibility	Public repository: GitHub (https://github.com) Repository name: pcm-dpc/COVID-19 Direct URL to data: https://github.com/pcm-dpc/COVID-19 License: CC-BY-4.0

## Value of the data

• These data are useful because they provide insight on the spread of SARS-CoV-2.
• The beneficiaries of these data are the general population and organizations, such as but not limited to governmental and health ones, who deal with SARS-CoV-2 spread worldwide.
• These data can be used: 1) to inform Italian and foreign citizens on the SARS-CoV-2 spread in Italy; 2) to support organizations in the evaluation of the efficiency of current prevention and control measures; and 3) to support governments in the future prevention decisions.
• The additional value of these data relies on the real-time (daily update) integrated surveillance of COVID-19 in Italy caused by SARS-CoV-2 and on their reliability due to their official source (Italian Civil Protection Department).

## 1 Data description

**Fig. 1:**
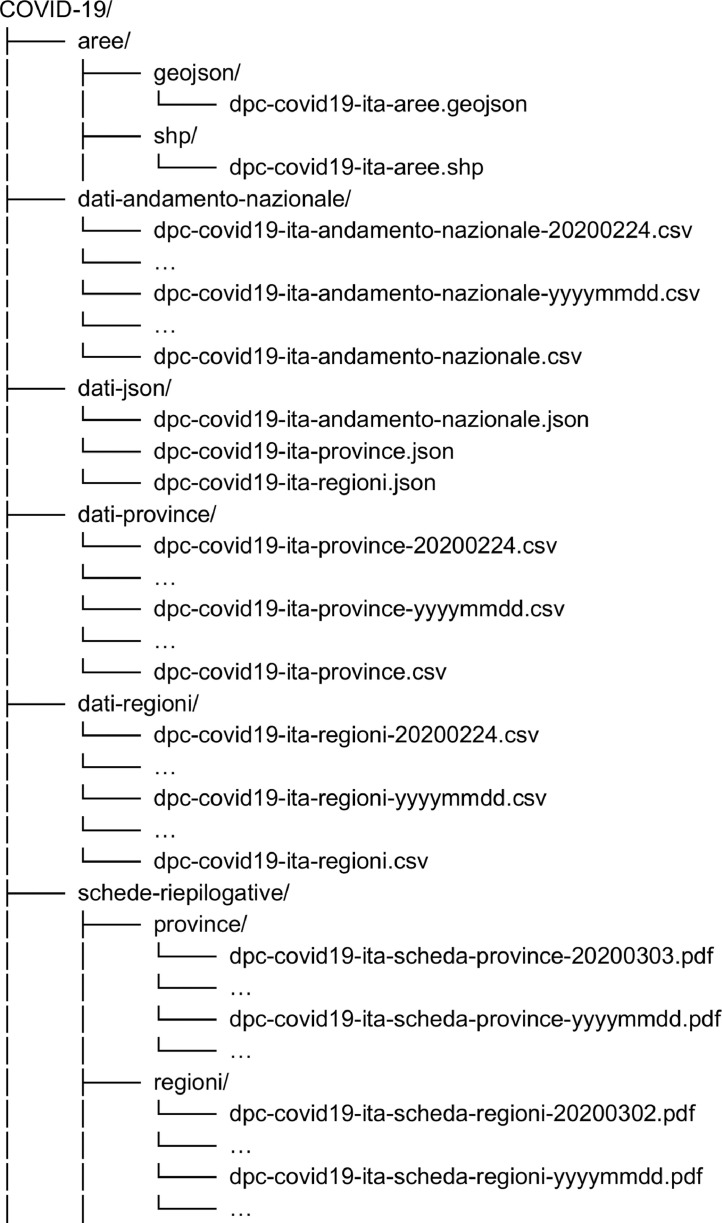
Physical structure of the COVID-19 database of the Italian Civil Protection Department.

The database here described contains data of integrated surveillance for the “Coronavirus disease 2019” (abbreviated as COVID-19 by the World Health Organization) in Italy, caused by the novel coronavirus called SARS-CoV-2 (Severe Acute Respiratory Sindrome-Coronavirus-2) by the International Committee on Taxonomy of Viruses. The database, included in a main folder called COVID-19 (Fig. 1), has been designed and created by the Italian Civil Protection Department, which currently manages it. The database consists of six folders (called ‘aree’, ‘dati-andamento-nazionale’, ‘dati-json’, ‘dati-province’, ‘dati-regioni’ and ‘schede-riepilogative’), the description of which is reported below. Under the main folder COVID-19 are also present other folders and files, useful for the database management and use, that are not further described in this document.

### 1.1 Aree

The folder called ‘aree’ contains charts of geographical areas interested by containment measures. It includes two subfolders, namely ‘geojson’ and ‘shp’, each containing a file called ‘dpc-covid19-ita-aree’ in two GIS formats, .geojson and .shp, respectively (the other three files present under ‘shp’ subfolder are mandatory for the .shp format).

### 1.2 Dati-andamento-nazionale

The folder called ‘dati-andamento-nazionale’ contains data relating to the national trend of SARS-CoV-2 spread. In particular, such data are organized into .csv separate daily files called ‘dpc-covid19-ita-andamento-nazionale-yyyymmdd’ (where yyyy is the year, mm is the month and dd is the day) as well as in a .csv cumulative file (one row per day) called ‘dpc-covid19-ita-andamento-nazionale’. Inside each file, data are structured in the 12 fields (one column per field) reported in Table 1. Specifically, Table 1 contains the structure of data (fields) contained in the .csv files inside the ‘dati-andamento-nazionale’ folder together with their description and format.

**Table 1:** Structure of data contained in the .csv files inside the ‘dati-andamento-nazionale’ folder.

Field name	Data description	Data format
data	notification date	yyyy-mm-dd hh:mm:ss CET (ISO 8601)
stato	country 3-letter code	XXX (ISO 3166-1 alpha-3)
ricoverati_con_sintomi	hospitalized patients with symptoms	integer number
terapia_intensiva	hospitalized patients in intensive care	integer number
totale_ospedalizzati	total hospitalized patients (hospitalized patients with symptoms + hospitalized patients in intensive care)	integer number
isolamento_domiciliare	home-confinement patients	integer number
totale_attualmente_positivi	total amount of currently positive cases (total hospitalized patients + home-confinement patients)	integer number
nuovi_attualmente_positivi	total amount of new positive cases (total amount of currently positive cases - total amount of positive cases of the previous day)	integer number
dimessi_guariti	recovered cases	integer number
deceduti	death	integer number
totale_casi	total amount of positive cases (total amount of currently positive cases + recovered cases + death)	integer number
tamponi	tests performed	integer number

### 1.3 Dati-json

The folder called ‘dati-json’ contains data that summarize the national, provincial and regional trends of SARS-CoV-2 spread. In particular, it includes three .json files, namely ‘dpc-covid19-ita-andamento-nazionale’, ‘dpc-covid19-ita-province’ and ‘dpc-covid19-ita-regioni’, that contain the same information of the homonymous .csv files under ‘dati-andamento-nazionale’,‘dati-province’ and ‘dati-regioni’ folders.

### 1.4 Dati-province

The folder called ‘dati-province’ contains data relating to the provincial trend of SARS-CoV-2 spread. In particular, such data are organized into .csv separate daily files called ‘dpc-covid19-ita-province-yyyymmdd’ (where yyyy is the year, mm is the month and dd is the day) as well as in a .csv cumulative file (one row per day) called ‘dpc-covid19-ita-province’. Inside each file, data are structured in the 10 fields (one column per field) reported in Table 2. Specifically, Table 2 reports the structure of data (fields) contained in the .csv files inside the ‘dati-province’ folder together with their description and format.

**Table 2:** Structure of data contained in the .csv files inside the ‘dati-province’ folder.

Field name	Data description	Data format
data	notification date	yyyy-mm-dd hh:mm:ss CET (ISO 8601)
stato	country 3-letter code	XXX (ISO 3166-1 alpha-3)
codice_regione	region 2-digit code	00 (ISTAT 2019)
denominazione_regione*	region name	text
codice_provincia§	province 3-digit code	000 (ISTAT 2019)
denominazione_provincia§	province name	text
sigla_provincia	province 2-letter code	XX
lat	latitude	decimal number (WGS84)
long	longitude	decimal number (WGS84)
totale_casi	total amount of positive cases	integer number

⁎ **denominazione_regione** of Trento and Bolzano, which are autonomous provinces, are indicated with “P.A. Trento” and “P.A. Bolzano”, respectively, instead of “Trentino Alto Adige”;

§ Rows in which **denominazione_provincia** is “In fase di definizione/aggiornamento" and **codice_provincia** is between 979 and 999, indicate data not assigned to any province yet.

### 1.5 Dati-regioni

The folder called ‘dati-regioni’ contains data relating to the regional trend of SARS-CoV-2 spread. In particular, such data are organized into .csv separate daily files called ‘dpc-covid19-ita-regioni-yyyymmdd’ (where yyyy is the year, mm is the month and dd is the day) as well as in a .csv cumulative file (one row per day) called ‘dpc-covid19-ita-regioni’. Inside each file, data are structured in the 16 fields (one column per field) reported in Table 3. Specifically, Table 3 reports the structure of data (fields) contained in the .csv files inside the ‘dati-regioni’ folder together with their description and format.

**Table 3:** Structure of data contained in the .csv files inside the ‘dati-regioni’ folder.

Field name	Data description	Data format
data	notification date	yyyy-mm-dd hh:mm:ss CET (ISO 8601)
stato	country 3-letter code	XXX (ISO 3166-1 alpha-3)
codice_regione	region 2-digit code	00 (ISTAT 2019)
denominazione_regione*	region name	text
lat	latitude	decimal number (WGS84)
long	longitude	decimal number (WGS84)
ricoverati_con_sintomi	hospitalized patients with symptoms	integer number
terapia_intensiva	hospitalized patients in intensive care	integer number
totale_ospedalizzati	total hospitalized patients (hospitalized patients with symptoms + hospitalized patients in intensive care)	integer number
isolamento_domiciliare	home-confinement patients	integer number
totale_attualmente_positivi	total amount of currently positive cases (total hospitalized patients + home-confinement patients)	integer number
nuovi_attualmente_positivi	total amount of new positive cases (total amount of currently positive cases - total amount of positive cases of the previous day)	integer number
dimessi_guariti	recovered cases	integer number
deceduti	death	integer number
totale_casi	total amount of positive cases (total amount of currently positive cases + recovered cases+ death)	integer number
tamponi	tests performed	integer number

⁎ **denominazione_regione** of Trento and Bolzano, which are autonomous provinces, are indicated with “P.A. Trento” and “P.A. Bolzano”, respectively, instead of “Trentino Alto Adige”

### 1.6 Schede-riepilogative

The folder called ‘schede-riepilogative’ contains summary sheets relating to the provincial and regional trends of SARS-CoV-2 spread. In particular, such sheets are organized into two subfolders, namely ‘province’ and ‘regioni’, each containing .pdf separate daily files called ‘dpc-covid19-ita-scheda-province-yyyymmdd’ and ‘dpc-covid19-ita-scheda-regioni-yyyymmdd’, respectively (where yyyy is the year, mm is the month and dd is the day).

The ‘dpc-covid19-ita-scheda-province-yyyymmdd’ files report the daily distribution, with date and time, of the positive cases over the regions and provinces (i.e. **Covid19 – Ripartizione dei contagiati per provincia al DD/MM/YYYY ore HH**). They contain several tables; each table represents a region and includes all the provinces of the region with the associated number of positive cases. Total number of positive cases per region is also provided. Sentences like ‘in fase di verifica e aggiornamento’, ‘in fase di verifica’, ‘in aggiornamento’, ‘in fase di aggiornamento’, ‘da aggiornare’ or similar indicate data pertaining to the region but not assigned to any province yet.

The ‘dpc-covid19-ita-scheda-regioni-yyyymmdd’ files report the daily update, with date and time (i.e. **AGGIORNAMENTO del DD/MM/YYYY ORE HH.MM**), of the distribution of the positive cases (i.e. **POSITIVI AL nCoV),** highlighted in yellow and separated as hospitalized patients with symptoms (i.e. **Ricoverati con sintomi**), hospitalized patients in intensive care (i.e. **Terapia intensiva**), home-confinement patients (i.e. **Isolamento domiciliare**) and total amount of currently positive cases (i.e. **Totale attualmente positivi**); the recovered cases (i.e. **DIMESSI GUARITI**) highlighted in green; the deaths (i.e. **DECEDUTI**) highlighted in red; the total amount of positive cases (i.e. **CASI TOTALI**) highlighted in orange; and the tests performed (i.e. **TAMPONI**).

## 2 Experimental design, materials, and methods

On January 31^st^, 2020, the Italian Council of Ministers declared a 6-month state of emergency as a consequence of the health risk associated with SARS-CoV-2 infection in Italy. Dr. Angelo Borrelli, being the Head of the Italian Civil Protection Department, is entrusted with the coordination of the interventions necessary to face the emergency on the national territory. His main responsibility is to coordinate actions aimed to rescue and assist the Italian population potentially affected by the infection, to strength controls in airport and port areas (in continuity with the urgent measures already adopted by the Italian Ministry of Health), and to support repatriation of both Italian citizens from foreign countries at risk and foreign citizen to their countries of origin. To inform citizens and make the collected data available (CC-BY-4.0 license), the Italian Civil Protection Department has developed an interactive geographic dashboard accessible at the addresses http://arcg.is/C1unv (desktop version) and http://arcg.is/081a51 (mobile version) which link the database described here (https://github.com/pcm-dpc/COVID-19).

The Italian Civil Protection Department daily receives data in a formal way by email. Epidemiological data about SARS-CoV-2 are daily collected by the regional institutions that send them to the Italian Ministry of Health. The Italian Ministry of Health, in turn, sends the data to the Italian Civil Protection Department. Consequently, the database is subject to daily updates and integrations which usually occur at about 6.30 pm CET.

## Appendix Supplementary materials

Image, application 1

Image, application 2

