# Supplementary material for: COVID-19 in Italy: Dataset of the Italian Civil Protection Department
Source: Data Brief. 2020 Apr 10;30:105526. doi: 10.1016/j.dib.2020.105526 (PMC7178485; doi:10.1016/j.dib.2020.105526)
Supplement: Supplementary file 2 [file mmc2.zip › COVID-19/schede-riepilogative/province/dpc-covid19-ita-scheda-province-20200303.pdf]

Covid 19 - Ripartizione dei contagiati per provincia al 03/03/2020 ore 17

LOMBARDIA	
Bergamo	372
Lodi	482
Cremona	287
in fase di verifica e aggiornamento	36
Pavia	122
Brescia	86
Milano	93
Monza Brianza	9
Mantova	15
Varese	7
Sondrio	3
Como	4
Lecco	4
Totale	***1520***

EMILIA-ROMAGNA	
Piacenza	256
Parma	84
Modena	33
Rimini	24
Reggio Emilia	14
Bologna	6
Ravenna	2
Forlì Cesena	1
Ferrara	0
Totale	***420***

VENETO	
PADOVA	144
TREVISO	82
VENEZIA	48
VERONA	17
in fase di verifica e aggiornamento	5
VICENZA	5
BELLUNO	5
ROVIGO	1
Totale	***307***

PIEMONTE	
Torino	7
Novara	3
Asti	40
Vercelli	1
Alessandria	1
Verbano-Cusio-Ossola	4
Totale	***56***

MARCHE	

Pesaro	55
Ancona	5
Macerata	1
Totale	***61***

LIGURIA	
Savona	20
Imperia	2
Genova	1
La Spezia	1
Totale	***24***

CAMPANIA	
Napoli	30
Totale	***30***

TOSCANA	
Firenze	7
Siena	3
Massa Carrara	3
Pistoia	1
Lucca	3
Arezzo	2
Totale	***19***

FRIULI VENEZIA GIULIA	
Trieste	2
Gorizia	3
Udine	8
Totale	***13***

LAZIO	
Roma	14
Totale	***14***

SICILIA	
Palermo	3
Sicilia da aggiornare	3
Catania	1
Totale	***7***

ABRUZZO	
Teramo	3
Pescara	1
L'aquila	1
Abruzzo da verificare	1
Totale	***6***

PUGLIA	
Taranto	3
Bari	2

Foggia	1
Totale	***6***
**UMBRIA**	
Perugia	1
Terni	1
Umbria da verificare	6
Totale	***8***
**TRENTINO ALTO ADIGE**	
Bolzano	1
Trento	4
Totale	***5***
**CALABRIA**	
Cosenza	1
Totale	***1***
**MOLISE**	
Campobasso	3
Totale	***3***
**BASILICATA**	
Potenza	1
Totale	***1***
**SARDEGNA**	
Cagliari	1
Totale	***1***
**Totale Generale**	**2502**
