# Supplementary material for: COVID-19 in Italy: Dataset of the Italian Civil Protection Department
Source: Data Brief. 2020 Apr 10;30:105526. doi: 10.1016/j.dib.2020.105526 (PMC7178485; doi:10.1016/j.dib.2020.105526)
Supplement: Supplementary file 2 [file mmc2.zip › COVID-19/schede-riepilogative/province/dpc-covid19-ita-scheda-province-20200304.pdf]

Covid 19 - Ripartizione dei contagiati per provincia al 04/03/2020 ore 17

LOMBARDIA	
Bergamo	423
Lodi	559
Cremona	333
in fase di verifica e aggiornamento	49
Pavia	126
Brescia	127
Milano	145
Monza Brianza	11
Mantova	22
Varese	11
Sondrio	4
Como	5
Lecco	5
Totale	***1820***

EMILIA-ROMAGNA	
Piacenza	319
Parma	115
Modena	41
Rimini	33
Reggio Emilia	20
Bologna	11
Ravenna	2
Forlì Cesena	2
Ferrara	0
in fase di verifica e aggiornamento	1
Totale	***544***

VENETO	
PADOVA	162
TREVISO	86
VENEZIA	59
VERONA	21
in fase di verifica e aggiornamento	11
VICENZA	10
BELLUNO	7
ROVIGO	4
Totale	***360***

PIEMONTE	
Torino	11
Novara	3
Asti	41
Vercelli	3
Alessandria	16
Verbano-Cusio-Ossola	5
in fase di verifica e aggiornamento	3

Totale	***82***

MARCHE	
Pesaro	72
Ancona	9
Macerata	2
Fermo	1
Totale	***84***

LIGURIA	
Savona	19
Imperia	2
Genova	1
La Spezia	1
Liguria da aggiornare	3
Totale	***26***

CAMPANIA	
Napoli	17
Campania da aggiornare	14
Totale	***31***

TOSCANA	
Firenze	12
Siena	10
Massa Carrara	4
Pistoia	1
Lucca	3
Arezzo	2
Pisa	2
Livorno	3
Prato	1
Totale	***38***

FRIULI VENEZIA GIULIA	
Trieste	5
Gorizia	5
Udine	8
Totale	***18***

LAZIO	
Roma	29
Frosinone	1
Totale	***30***

SICILIA	
Palermo	3
Sicilia da aggiornare	14
Catania	1
Totale	***18***

ABRUZZO	
Teramo	3
Pescara	1
L'aquila	1
Abruzzo da verificare	2
Totale	***7***

PUGLIA	
Taranto	3
Bari	2
Brindisi	
Bat	1
Lecce	1
Foggia	2
Totale	***9***

UMBRIA	
Perugia	6
Terni	3
Totale	***9***

TRENTINO ALTO ADIGE	
Bolzano	1
Trento	5
Totale	***6***

CALABRIA	
Cosenza	1
Totale	***1***

MOLISE	
Campobasso	3
Totale	***3***

BASILICATA	
Potenza	1
Totale	***1***

SARDEGNA	
Cagliari	2
Totale	***2***

Totale Generale	3089
