# Supplementary material for: COVID-19 in Italy: Dataset of the Italian Civil Protection Department
Source: Data Brief. 2020 Apr 10;30:105526. doi: 10.1016/j.dib.2020.105526 (PMC7178485; doi:10.1016/j.dib.2020.105526)
Supplement: Supplementary file 2 [file mmc2.zip › COVID-19/schede-riepilogative/province/dpc-covid19-ita-scheda-province-20200305.pdf]

Covid 19 - Ripartizione dei contagiati per provincia al 05/03/2020 ore 17

LOMBARDIA	
Bergamo	537
Lodi	658
Cremona	406
in fase di verifica e aggiornamento	62
Pavia	151
Brescia	155
Milano	197
Monza Brianza	19
Mantova	26
Varese	17
Sondrio	4
Como	11
Lecco	8
Totale	***2251***

EMILIA-ROMAGNA	
Piacenza	378
Parma	150
Modena	45
Rimini	68
Reggio Emilia	31
Bologna	19
Ravenna	3
Forlì Cesena	4
Ferrara	0
in fase di verifica e aggiornamento	
Totale	***698***

VENETO	
PADOVA	175
TREVISO	89
VENEZIA	73
VERONA	25
in fase di verifica e aggiornamento	14
VICENZA	19
BELLUNO	7
ROVIGO	5
Totale	***407***

PIEMONTE	
Torino	19
Novara	3
Asti	43
Vercelli	8
Alessandria	22
Verbano-Cusio-Ossola	5
BIELLA	2
in fase di verifica e aggiornamento	6

Totale	***108***
**MARCHE**	
Pesaro	100
Ancona	19
Macerata	2
Fermo	3
Totale	***124***
**LIGURIA**	
Savona	20
Imperia	2
Genova	1
La Spezia	1
Liguria da aggiornare	4
Totale	***28***
**CAMPANIA**	
Napoli	17
Campania da aggiornare	28
Totale	***45***
**TOSCANA**	
Firenze	20
Siena	12
Massa Carrara	6
Pistoia	1
Lucca	6
Arezzo	5
Pisa	5
Livorno	3
Prato	1
Grosseto	2
Totale	***61***
**FRIULI VENEZIA GIULIA**	
Trieste	5
Gorizia	5
Udine	11
Totale	***21***
**LAZIO**	
Roma	42
Frosinone	1
Lazio in fase di verifica	1
Totale	***44***
**SICILIA**	
Palermo	3
Sicilia da aggiornare	14
Catania	1

Totale	***18***
**ABRUZZO**	
Teramo	4
Pescara	1
L'aquila	1
Chieti	2
Totale	***8***
**PUGLIA**	
Taranto	3
Bari	2
Brindisi	
Bat	1
Lecce	1
Foggia	7
Totale	***14***
**UMBRIA**	
Perugia	6
Terni	3
Totale	***9***
**TRENTINO ALTO ADIGE**	
Bolzano	1
Trento	7
Totale	***8***
**CALABRIA**	
Cosenza	1
Catanzaro	1
Totale	***2***
**MOLISE**	
Campobasso	7
Totale	***7***
**BASILICATA**	
Potenza	1
Totale	***1***
**SARDEGNA**	
Cagliari	2
Totale	***2***
**VALLE D'AOSTA**	
AOSTA	2
Totale	***2***
**Totale Generale**	**3858**
