# Supplementary material for: COVID-19 in Italy: Dataset of the Italian Civil Protection Department
Source: Data Brief. 2020 Apr 10;30:105526. doi: 10.1016/j.dib.2020.105526 (PMC7178485; doi:10.1016/j.dib.2020.105526)
Supplement: Supplementary file 2 [file mmc2.zip › COVID-19/schede-riepilogative/province/dpc-covid19-ita-scheda-province-20200306.pdf]

Covid 19 - Ripartizione dei contagiati per provincia al 06/03/2020 ore 17

LOMBARDIA	
Bergamo	623
Lodi	739
Cremona	452
in fase di verifica e aggiornamento	68
Pavia	180
Brescia	182
Milano	267
Monza Brianza	20
Mantova	32
Varese	23
Sondrio	4
Como	11
Lecco	11
Totale	***2612***

EMILIA-ROMAGNA	
Piacenza	426
Parma	181
Modena	73
Rimini	93
Reggio Emilia	44
Bologna	41
Ravenna	8
Forlì Cesena	3
Ferrara	1
in fase di verifica e aggiornamento	
Totale	***870***

VENETO	
PADOVA	198
TREVISO	103
VENEZIA	85
VERONA	42
in fase di verifica e aggiornamento	24
VICENZA	24
BELLUNO	7
ROVIGO	5
Totale	***488***

MARCHE	
Pesaro	126
Ancona	23
Macerata	7
Fermo	3
Totale	***159***

PIEMONTE	
Torino	34

Novara	4
Asti	47
Vercelli	7
Alessandria	32
Verbano-Cusio-Ossola	9
BIELLA	3
CUNEO	1
in fase di verifica e aggiornamento	6
Totale	***143***

TOSCANA	
Firenze	23
Siena	14
Massa Carrara	10
Pistoia	1
Lucca	8
Arezzo	7
Pisa	8
Livorno	5
Prato	1
Grosseto	2
Totale	***79***

CAMPANIA	
Napoli	17
Campania da aggiornare	40
Totale	***57***

LAZIO	
Roma	49
Frosinone	1
Viterbo	2
Latina	2
Totale	***54***

LIGURIA	
Savona	15
Imperia	3
Genova	9
La Spezia	1
Altro/in fase di verifica	4
Totale	***32***

FRIULI VENEZIA GIULIA	
Trieste	7
Gorizia	6
Udine	18
Totale	***31***

SICILIA	
Palermo	5

Enna	1
Catania	15
Ragusa	1
Siracusa	2
Totale	***24***
**PUGLIA**	
Taranto	3
Bari	3
Brindisi	
Bat	1
Lecce	3
Foggia	7
Totale	***17***
**UMBRIA**	
Perugia	10
Terni	6
Totale	***16***
**ABRUZZO**	
Teramo	4
Pescara	1
L'aquila	1
Chieti	3
Totale	***9***
**MOLISE**	
Campobasso	12
Totale	***12***
**TRENTINO ALTO ADIGE**	
Bolzano	4
Trento	10
Totale	***14***
**SARDEGNA**	
Cagliari	3
Nuoro	2
Totale	***5***
**BASILICATA**	
Potenza	2
Matera	1
Totale	***3***
**VALLE D'AOSTA**	
AOSTA	7
Totale	***7***
**CALABRIA**	

Cosenza	1
Reggio Calabria	1
Catanzaro	2
Totale	***4***
**Totale Generale**	**4636**
