# Supplementary material for: COVID-19 in Italy: Dataset of the Italian Civil Protection Department
Source: Data Brief. 2020 Apr 10;30:105526. doi: 10.1016/j.dib.2020.105526 (PMC7178485; doi:10.1016/j.dib.2020.105526)
Supplement: Supplementary file 2 [file mmc2.zip › COVID-19/schede-riepilogative/province/dpc-covid19-ita-scheda-province-20200307.pdf]

Covid 19 - Ripartizione dei contagiati per provincia al 07/03/2020 ore 17

LOMBARDIA	
Bergamo	761
Lodi	811
Cremona	562
in fase di verifica e aggiornamento	93
Pavia	221
Brescia	413
Milano	361
Monza Brianza	61
Mantova	46
Varese	27
Sondrio	6
Como	23
Lecco	35
Totale	***3420***

EMILIA-ROMAGNA	
Piacenza	479
Parma	229
Modena	82
Rimini	104
Reggio Emilia	48
Bologna	49
Ravenna	10
Forlì Cesena	7
Ferrara	2
in fase di verifica e aggiornamento	
Totale	***1010***

VENETO	
PADOVA	216
TREVISO	110
VENEZIA	100
VERONA	52
in fase di verifica e aggiornamento	12
VICENZA	37
BELLUNO	11
ROVIGO	5
Totale	***543***

MARCHE	
Pesaro	159
Ancona	38
Macerata	7
Fermo	3
Totale	***207***

PIEMONTE	
Torino	55

Novara	5
Asti	50
Vercelli	10
Alessandria	40
Verbano-Cusio-Ossola	10
BIELLA	6
CUNEO	1
in fase di verifica e aggiornamento	30
Totale	***207***

TOSCANA	
Firenze	27
Siena	19
Massa Carrara	16
Pistoia	6
Lucca	15
Arezzo	8
Pisa	11
Livorno	6
Prato	3
Grosseto	2
Totale	***113***

CAMPANIA	
Napoli	17
Campania da aggiornare	44
Totale	***61***

LAZIO	
Roma	71
Frosinone	1
Viterbo	2
Latina	2
Totale	***76***

LIGURIA	
Savona	19
Imperia	7
Genova	15
La Spezia	5
Altro/in fase di verifica	5
Totale	***51***

FRIULI VENEZIA GIULIA	
Trieste	11
Gorizia	6
Udine	23
Pordenone	2
Totale	***42***

SICILIA	

Palermo	6
Enna	
Catania	23
Ragusa	1
Agrigento	1
Messina	2
Siracusa	2
Totale	***35***
**PUGLIA**	
Taranto	3
Bari	5
Brindisi	3
Bat	2
Lecce	4
Foggia	9
Totale	***26***
**UMBRIA**	
Perugia	15
Terni	9
Totale	***24***
**ABRUZZO**	
Teramo	4
Pescara	2
L'aquila	1
Chieti	4
Totale	***11***
**MOLISE**	
Campobasso	14
Totale	***14***
**TRENTINO ALTO ADIGE**	
Bolzano	9
Trento	14
Totale	***23***
**SARDEGNA**	
Cagliari	3
Nuoro	2
Totale	***5***
**BASILICATA**	
Potenza	2
Matera	1
Totale	***3***
**VALLE D'AOSTA**	
AOSTA	8

Totale	***8***
**CALABRIA**	
Cosenza	1
Reggio Calabria	1
Catanzaro	2
Totale	***4***
**Totale Generale**	**5883**
