# Supplementary material for: COVID-19 in Italy: Dataset of the Italian Civil Protection Department
Source: Data Brief. 2020 Apr 10;30:105526. doi: 10.1016/j.dib.2020.105526 (PMC7178485; doi:10.1016/j.dib.2020.105526)
Supplement: Supplementary file 2 [file mmc2.zip › COVID-19/schede-riepilogative/province/dpc-covid19-ita-scheda-province-20200308.pdf]

Covid 19 - Ripartizione dei contagiati per provincia al 08/03/2020 ore 17

LOMBARDIA	
Bergamo	997
Lodi	853
Cremona	665
in fase di verifica e aggiornamento	291
Pavia	243
Brescia	501
Milano	406
Monza Brianza	59
Mantova	56
Varese	32
Sondrio	6
Como	27
Lecco	53
Totale	***4189***

EMILIA-ROMAGNA	
Piacenza	528
Parma	276
Modena	97
Rimini	113
Reggio Emilia	70
Bologna	62
Ravenna	13
Forlì Cesena	15
Ferrara	6
in fase di verifica e aggiornamento	
Totale	***1180***

VENETO	
PADOVA	255
TREVISO	126
VENEZIA	126
VERONA	63
in fase di verifica e aggiornamento	22
VICENZA	50
BELLUNO	23
ROVIGO	5
Totale	***670***

MARCHE	
Pesaro	204
Ancona	54
Macerata	9
Fermo	5
Totale	***272***

PIEMONTE	
Torino	89

Novara	13
Asti	58
Vercelli	15
Alessandria	60
Verbano-Cusio-Ossola	13
BIELLA	19
CUNEO	5
in fase di verifica e aggiornamento	88
Totale	***360***

TOSCANA	
Firenze	39
Siena	24
Massa Carrara	21
Pistoia	13
Lucca	26
Arezzo	9
Pisa	17
Livorno	10
Prato	3
Grosseto	4
Totale	***166***

CAMPANIA	
Napoli	45
Salerno	15
caserta	28
avellino	3
benevento	4
In aggiornamento	6
Totale	***101***

LAZIO	
Roma	77
Frosinone	2
Viterbo	2
Latina	6
Totale	***87***

LIGURIA	
Savona	25
Imperia	10
Genova	25
La Spezia	11
in fase di aggiornamento	7
Totale	***78***

FRIULI VENEZIA GIULIA	
Trieste	25
Gorizia	6
Udine	24

Pordenone	2
Totale	***57***

SICILIA	
Palermo	6
Enna	
Catania	23
Ragusa	1
Agrigento	1
Messina	2
Siracusa	2
in fase di aggiornamento	18
Totale	***53***

PUGLIA	
Taranto	3
Bari	5
Brindisi	3
Bat	3
Lecce	10
Foggia	16
Totale	***40***

UMBRIA	
Perugia	15
Terni	11
Totale	***26***

ABRUZZO	
Teramo	4
Pescara	8
L'aquila	1
Chieti	4
Totale	***17***

MOLISE	
Campobasso	14
Totale	***14***

TRENTINO ALTO ADIGE	
Bolzano	9
Trento	23
Totale	***32***

SARDEGNA	
Cagliari	9
Nuoro	2
Totale	***11***

BASILICATA	
Potenza	2

Matera	2
Totale	***4***
**VALLE D'AOSTA**	
AOSTA	9
Totale	***9***
**CALABRIA**	
Cosenza	1
Reggio Calabria	1
Catanzaro	2
altro in fase di aggiornamento	5
Totale	***9***
**Totale Generale**	**7375**
