# Supplementary material for: COVID-19 in Italy: Dataset of the Italian Civil Protection Department
Source: Data Brief. 2020 Apr 10;30:105526. doi: 10.1016/j.dib.2020.105526 (PMC7178485; doi:10.1016/j.dib.2020.105526)
Supplement: Supplementary file 2 [file mmc2.zip › COVID-19/schede-riepilogative/province/dpc-covid19-ita-scheda-province-20200309.pdf]

Covid 19 - Ripartizione dei contagiati per provincia al 09/03/2020 ore 17

LOMBARDIA	
Bergamo	1245
Lodi	928
Cremona	916
in fase di verifica e aggiornamento	516
Pavia	296
Brescia	739
Milano	506
Monza Brianza	64
Mantova	102
Varese	44
Sondrio	7
Como	40
Lecco	66
Totale	***5469***

EMILIA-ROMAGNA	
Piacenza	602
Parma	279
Modena	116
Rimini	164
Reggio Emilia	103
Bologna	80
Ravenna	19
Forlì Cesena	16
Ferrara	7
in fase di verifica e aggiornamento	
Totale	***1386***

VENETO	
PADOVA	273
TREVISO	136
VENEZIA	130
VERONA	73
in fase di verifica e aggiornamento	43
VICENZA	53
BELLUNO	29
ROVIGO	7
Totale	***744***

MARCHE	
Pesaro	246
Ancona	63
Macerata	9
Fermo	5
Totale	***323***

PIEMONTE	
Torino	87

Novara	14
Asti	58
Vercelli	11
Alessandria	63
Verbano-Cusio-Ossola	11
BIELLA	18
CUNEO	11
in fase di verifica e aggiornamento	77
Totale	***350***

TOSCANA	
Firenze	51
Siena	29
Massa Carrara	28
Pistoia	16
Lucca	31
Arezzo	12
Pisa	20
Livorno	10
Prato	5
Grosseto	6
Totale	***208***

CAMPANIA	
Napoli	55
Salerno	17
caserta	32
avellino	3
benevento	4
In aggiornamento	9
Totale	***120***

LAZIO	
Roma	91
Frosinone	2
Viterbo	2
Latina	6
Lazio in aggiornamento	1
Totale	***102***

LIGURIA	
Savona	35
Imperia	11
Genova	38
La Spezia	15
in fase di aggiornamento	10
Totale	***109***

FRIULI VENEZIA GIULIA	
Trieste	25
Gorizia	6

Udine	24
Pordenone	2
Friuli in aggiornamento	36
Totale	***93***
**SICILIA**	
Palermo	6
Enna	
Catania	23
Ragusa	1
Agrigento	1
Messina	2
Siracusa	2
in fase di aggiornamento	19
Totale	***54***
**PUGLIA**	
Taranto	3
Bari	6
Brindisi	5
Bat	3
Lecce	10
Foggia	23
Totale	***50***
**UMBRIA**	
Perugia	12
Terni	16
Totale	***28***
**ABRUZZO**	
Teramo	4
Pescara	14
L'aquila	5
Chieti	7
Totale	***30***
**MOLISE**	
Campobasso	14
Totale	***14***
**TRENTINO ALTO ADIGE**	
Bolzano	9
Trento	33
Totale	***42***
**SARDEGNA**	
Cagliari	14
Nuoro	3
Oristano	1
Sassari	1

Totale	***19***
**BASILICATA**	
Potenza	2
Matera	3
Totale	***5***
**VALLE D'AOSTA**	
AOSTA	15
Totale	***15***
**CALABRIA**	
Cosenza	4
Reggio Calabria	2
Catanzaro	3
Vibo Valentia	2
Totale	***11***
**Totale Generale**	**9172**
