# Supplementary material for: COVID-19 in Italy: Dataset of the Italian Civil Protection Department
Source: Data Brief. 2020 Apr 10;30:105526. doi: 10.1016/j.dib.2020.105526 (PMC7178485; doi:10.1016/j.dib.2020.105526)
Supplement: Supplementary file 2 [file mmc2.zip › COVID-19/schede-riepilogative/province/dpc-covid19-ita-scheda-province-20200310.pdf]

Covid 19 - Ripartizione dei contagiati per provincia al 10/03/2020 ore 17

LOMBARDIA	
Bergamo	1472
Lodi	963
Cremona	957
in fase di verifica e aggiornamento	317
Pavia	324
Brescia	790
Milano	592
Monza Brianza	65
Mantova	119
Varese	50
Sondrio	7
Como	46
Lecco	89
Totale	***5791***

EMILIA-ROMAGNA	
Piacenza	633
Parma	325
Modena	127
Rimini	206
Reggio Emilia	104
Bologna	86
Ravenna	24
Forlì Cesena	20
Ferrara	8
in fase di verifica e aggiornamento	
Totale	***1533***

VENETO	
PADOVA	296
TREVISO	158
VENEZIA	152
VERONA	96
in fase di verifica e aggiornamento	42
VICENZA	73
BELLUNO	29
ROVIGO	10
Totale	***856***

MARCHE	
Pesaro	296
Ancona	81
Macerata	11
Fermo	6
Totale	***394***

PIEMONTE	
Torino	111

Novara	22
Asti	58
Vercelli	18
Alessandria	65
Verbano-Cusio-Ossola	11
BIELLA	20
CUNEO	14
in fase di verifica e aggiornamento	134
Totale	***453***

TOSCANA	
Firenze	61
Siena	33
Massa Carrara	37
Pistoia	21
Lucca	37
Arezzo	13
Pisa	31
Livorno	14
Prato	7
Grosseto	10
Totale	***264***

CAMPANIA	
Napoli	61
Salerno	17
Caserta	34
Avellino	3
Benevento	4
In aggiornamento	8
Totale	***127***

LAZIO	
Roma	76
Frosinone	6
Viterbo	5
Rieti	1
Latina	11
Lazio Fuori Regione	17
Totale	***116***

LIGURIA	
Savona	38
Imperia	15
Genova	42
La Spezia	15
in fase di aggiornamento	31
Totale	***141***

FRIULI VENEZIA GIULIA	
Trieste	53

Gorizia	10
Udine	26
Pordenone	4
Friuli in aggiornamento	23
Totale	***116***

**SICILIA**	
Palermo	10
Enna	1
Catania	35
Ragusa	1
Agrigento	11
Messina	2
Siracusa	2
Totale	***62***

**PUGLIA**	
Taranto	3
Bari	15
Brindisi	5
Bat	3
Lecce	10
Foggia	23
Totale	***59***

**UMBRIA**	
Perugia	13
Terni	24
Totale	***37***

**ABRUZZO**	
Teramo	5
Pescara	18
L'aquila	6
Chieti	9
Totale	***38***

**MOLISE**	
Campobasso	15
Totale	***15***

**TRENTINO ALTO ADIGE**	
Bolzano	38
Trento	52
Totale	***90***

**SARDEGNA**	
Cagliari	15
Nuoro	3
Oristano	1
Sassari	1

Totale	***20***
**BASILICATA**	
Potenza	4
Matera	3
Totale	***7***
**VALLE D'AOSTA**	
AOSTA	17
Totale	***17***
**CALABRIA**	
Cosenza	6
Reggio Calabria	3
Catanzaro	3
Vibo Valentia	1
Totale	***13***
**Totale Generale**	**10149**
