# Supplementary material for: COVID-19 in Italy: Dataset of the Italian Civil Protection Department
Source: Data Brief. 2020 Apr 10;30:105526. doi: 10.1016/j.dib.2020.105526 (PMC7178485; doi:10.1016/j.dib.2020.105526)
Supplement: Supplementary file 2 [file mmc2.zip › COVID-19/schede-riepilogative/province/dpc-covid19-ita-scheda-province-20200311.pdf]

Covid 19 - Ripartizione dei contagiati per provincia al 11/03/2020 ore 17

LOMBARDIA	
Bergamo	1815
Lodi	1035
Cremona	1061
in fase di verifica e aggiornamento	190
Pavia	403
Brescia	1351
Milano	925
Monza Brianza	85
Mantova	137
Varese	75
Sondrio	13
Como	77
Lecco	113
Totale	***7280***

EMILIA-ROMAGNA	
Piacenza	664
Parma	378
Modena	163
Rimini	245
Reggio Emilia	114
Bologna	108
Ravenna	31
Forlì Cesena	24
Ferrara	12
in fase di verifica e aggiornamento	
Totale	***1739***

VENETO	
PADOVA	373
TREVISO	185
VENEZIA	179
VERONA	110
in fase di verifica e aggiornamento	40
VICENZA	92
BELLUNO	30
ROVIGO	14
Totale	***1023***

MARCHE	
Pesaro	342
Ancona	110
Macerata	17
Fermo	8
Ascoli Piceno	1
altro/in fase di verifica	1
Totale	***479***

PIEMONTE	
Torino	159
Novara	27
Asti	68
Vercelli	24
Alessandria	105
Verbano-Cusio-Ossola	13
BIELLA	36
CUNEO	17
in fase di verifica e aggiornamento	52
Totale	***501***

TOSCANA	
Firenze	71
Siena	37
Massa Carrara	40
Pistoia	32
Lucca	43
Arezzo	14
Pisa	34
Livorno	16
Prato	21
Grosseto	12
Totale	***320***

CAMPANIA	
Napoli	96
Salerno	17
Caserta	26
Avellino	7
Benevento	2
In aggiornamento	6
Totale	***154***

LAZIO	
Roma	99
Frosinone	8
Viterbo	10
Rieti	3
Latina	11
Lazio Fuori Regione	19
Totale	***150***

LIGURIA	
Savona	42
Imperia	18
Genova	63
La Spezia	19
in fase di aggiornamento	52
Totale	***194***

FRIULI VENEZIA GIULIA	
Trieste	57
Gorizia	12
Udine	44
Pordenone	13
Friuli in aggiornamento	
Totale	***126***

SICILIA	
Palermo	15
Enna	1
Catania	41
Ragusa	1
Agrigento	16
Messina	4
Siracusa	3
Trapani	2
Totale	***83***

PUGLIA	
Taranto	4
Bari	18
Brindisi	15
Bat	4
Lecce	12
Foggia	24
Totale	***77***

UMBRIA	
Perugia	26
Terni	18
Da aggiornare	2
Totale	***46***

ABRUZZO	
Teramo	5
Pescara	18
L'aquila	6
Chieti	9
Totale	***38***

MOLISE	
Campobasso	16
Totale	***16***

TRENTINO ALTO ADIGE	
Bolzano	75
Trento	77
Totale	***152***

SARDEGNA	

Cagliari	15
Nuoro	18
Oristano	2
Sassari	2
Totale	***37***
**BASILICATA**	
Potenza	5
Matera	3
Totale	***8***
**VALLE D'AOSTA**	
AOSTA	20
Totale	***20***
**CALABRIA**	
Cosenza	5
Reggio Calabria	6
Catanzaro	2
Vibo Valentia	5
Crotone	1
Totale	***19***
**Totale Generale**	**12462**
