# Supplementary material for: COVID-19 in Italy: Dataset of the Italian Civil Protection Department
Source: Data Brief. 2020 Apr 10;30:105526. doi: 10.1016/j.dib.2020.105526 (PMC7178485; doi:10.1016/j.dib.2020.105526)
Supplement: Supplementary file 2 [file mmc2.zip › COVID-19/schede-riepilogative/province/dpc-covid19-ita-scheda-province-20200312.pdf]

Covid 19 - Ripartizione dei contagiati per provincia al 12/03/2020 ore 17

LOMBARDIA	
Bergamo	2136
Lodi	1123
Cremona	1302
in fase di verifica e aggiornamento	235
Pavia	468
Brescia	1598
Milano	1146
Monza Brianza	130
Mantova	169
Varese	98
Sondrio	23
Como	98
Lecco	199
Totale	***8725***

EMILIA-ROMAGNA	
Piacenza	679
Parma	430
Modena	190
Rimini	312
Reggio Emilia	123
Bologna	122
Ravenna	41
Forlì Cesena	33
Ferrara	17
in fase di verifica e aggiornamento	
Totale	***1947***

VENETO	
PADOVA	439
TREVISO	279
VENEZIA	205
VERONA	150
in fase di verifica e aggiornamento	128
VICENZA	122
BELLUNO	48
ROVIGO	13
Totale	***1384***

MARCHE	
ANCONA	142
PESARO	403
MACERATA	32
FERMO	11
ASCOLI PICENO	1
altro/in fase di aggiornamento	3
Totale	***592***

PIEMONTE	
ALESSANDRIA	132
ASTI	69
BIELLA	39
CUNEO	24
Novara	32
Torino	187
VERCELLI	25
Verbano-Cusio-Ossola	18
altro/in fase di aggiornamento	54
Totale	***580***

TOSCANA	
Firenze	86
Siena	41
Massa Carrara	40
Pistoia	43
Lucca	49
Arezzo	14
Pisa	34
Livorno	16
Prato	25
Grosseto	16
Totale	***364***

CAMPANIA	
Napoli	109
Salerno	18
Caserta	29
Avellino	12
Benevento	3
In aggiornamento	8
Totale	***179***

LAZIO	
Roma	162
Frosinone	18
Viterbo	5
Rieti	2
Latina	11
Lazio Fuori Regione	2
Totale	***200***

LIGURIA	
Savona	52
Imperia	32
Genova	92
La Spezia	26
in fase di aggiornamento	72
Totale	***274***

FRIULI VENEZIA GIULIA	
Trieste	57
Gorizia	12
Udine	44
Pordenone	13
Friuli in aggiornamento	41
Totale	***167***

SICILIA	
Palermo	26
Enna	1
Catania	49
Ragusa	2
Agrigento	17
Messina	9
Siracusa	5
Trapani	4
Caltanisetta	2
Totale	***115***

PUGLIA	
BARI	25
BAT	6
BRINDISI	16
FOGGIA	33
LECCE	18
TARANTO	6
TOTALE	***104***

UMBRIA	
Perugia	37
Terni	25
Da aggiornare	2
Totale	***64***

ABRUZZO	
Teramo	8
Pescara	48
L'aquila	8
Chieti	20
Totale	***84***

MOLISE	
Campobasso	16
Totale	***16***

TRENTINO ALTO ADIGE	
Bolzano	104
Trento	107
Totale	***211***

SARDEGNA	
Città metropolitana di Cagiari	11
Sud Sardegna	4
Oristano	2
Nuoro	18
Sassari	4
Totale	***39***

BASILICATA	
Potenza	5
Matera	3
Totale	***8***

VALLE D'AOSTA	
AOSTA	27
Totale	***27***

CALABRIA	
Cosenza	8
Reggio Calabria	11
Catanzaro	3
Vibo Valentia	5
Crotone	6
Altro/In fase di aggiornamento	
Totale	***33***

**Totale Generale**	**15113**
