# Supplementary material for: COVID-19 in Italy: Dataset of the Italian Civil Protection Department
Source: Data Brief. 2020 Apr 10;30:105526. doi: 10.1016/j.dib.2020.105526 (PMC7178485; doi:10.1016/j.dib.2020.105526)
Supplement: Supplementary file 2 [file mmc2.zip › COVID-19/schede-riepilogative/province/dpc-covid19-ita-scheda-province-20200313.pdf]

Covid 19 - Ripartizione dei contagiati per provincia al 13/03/2020 ore 17

LOMBARDIA	
Bergamo	2368
Lodi	1133
Cremona	1344
in fase di verifica e aggiornamento	569
Pavia	482
Brescia	1784
Milano	1307
Monza Brianza	143
Mantova	187
Varese	125
Sondrio	23
Como	118
Lecco	237
Totale	***9820***

EMILIA-ROMAGNA	
Piacenza	710
Parma	518
Modena	251
Rimini	363
Reggio Emilia	138
Bologna	155
Ravenna	55
Forlì Cesena	49
Ferrara	24
in fase di verifica e aggiornamento	
Totale	***2263***

VENETO	
PADOVA	523
VENEZIA	248
VICENZA	141
VERONA	210
BELLUNO	59
ROVIGO	16
TREVISO	327
altro/in fase di verifica	71
Totale	***1595***

MARCHE	
ANCONA	158
PESARO	496
MACERATA	48
FERMO	16
ASCOLI PICENO	2
altro/in fase di aggiornamento	5
Totale	***725***

PIEMONTE	
ALESSANDRIA	136
ASTI	70
BIELLA	48
CUNEO	40
Novara	48
Torino	305
VERCELLI	29
Verbano-Cusio-Ossola	29
altro/in fase di aggiornamento	135
Totale	***840***

TOSCANA	
Firenze	101
Siena	41
Massa Carrara	65
Pistoia	48
Lucca	79
Arezzo	16
Pisa	51
Livorno	27
Prato	26
Grosseto	16
Totale	***470***

CAMPANIA	
Napoli	140
Salerno	20
Caserta	38
Avellino	14
Benevento	3
In aggiornamento	5
Totale	***220***

LAZIO	
Roma	218
Frosinone	28
Viterbo	10
Rieti	3
Latina	15
in fase di aggiornamento	3
Totale	***277***

LIGURIA	
Savona	62
Imperia	41
Genova	128
La Spezia	37
in fase di aggiornamento	77
Totale	***345***

FRIULI VENEZIA GIULIA	
Trieste	131
Gorizia	20
Udine	75
Pordenone	31
Friuli in aggiornamento	
Totale	***257***

SICILIA	
Palermo	26
Enna	1
Catania	49
Ragusa	2
Agrigento	17
Messina	9
Siracusa	5
Trapani	4
Caltanisetta	2
In aggiornamento	15
Totale	***130***

PUGLIA	
BARI	38
BAT	10
BRINDISI	20
FOGGIA	34
LECCE	19
TARANTO	8
TOTALE	***129***

UMBRIA	
Perugia	47
Terni	29
Da aggiornare	
Totale	***76***

ABRUZZO	
Teramo	9
Pescara	48
L'aquila	9
Chieti	23
Totale	***89***

MOLISE	
Campobasso	17
Totale	***17***

TRENTINO ALTO ADIGE	
Bolzano	125
Trento	163
Totale	***288***

SARDEGNA	
Città metropolitana di Cagliari	15
Sud Sardegna	4
Oristano	2
Nuoro	18
Sassari	4
Totale	***43***

BASILICATA	
Potenza	7
Matera	3
Totale	***10***

VALLE D'AOSTA	
AOSTA	28
Totale	***28***

CALABRIA	
Cosenza	10
Reggio Calabria	13
Catanzaro	3
Vibo Valentia	5
Crotone	7
Altro/In fase di aggiornamento	
Totale	***38***

**Totale Generale**	**17660**
