# Supplementary material for: COVID-19 in Italy: Dataset of the Italian Civil Protection Department
Source: Data Brief. 2020 Apr 10;30:105526. doi: 10.1016/j.dib.2020.105526 (PMC7178485; doi:10.1016/j.dib.2020.105526)
Supplement: Supplementary file 2 [file mmc2.zip › COVID-19/schede-riepilogative/province/dpc-covid19-ita-scheda-province-20200314.pdf]

Covid 19 - Ripartizione dei contagiati per provincia al 14/03/2020 ore 17

LOMBARDIA	
Bergamo	2864
Brescia	2122
Como	154
Cremona	1565
in fase di verifica e aggiornamento	556
Lecco	287
Lodi	1276
Mantova	261
Milano	1551
Monza Brianza	224
Pavia	622
Sondrio	45
Varese	158
Totale	***11685***

EMILIA-ROMAGNA	
Piacenza	853
Bologna	195
Ferrara	29
Forlì Cesena	62
in fase di verifica e aggiornamento	
Modena	306
Parma	570
Ravenna	78
Reggio Emilia	153
Rimini	398
Totale	***2644***

VENETO	
PADOVA	611
VENEZIA	282
VICENZA	164
VERONA	275
BELLUNO	78
ROVIGO	27
TREVISO	376
altro/in fase di verifica	124
Totale	***1937***

MARCHE	
ANCONA	215
PESARO	591
MACERATA	58
FERMO	22
ASCOLI PICENO	7
altro/in fase di verifica	6
Totale	***899***

PIEMONTE	
ALESSANDRIA	182
ASTI	87
BIELLA	48
CUNEO	47
Novara	48
Torino	305
VERCELLI	31
Verbano-Cusio-Ossola	36
altro/in fase di aggiornamento	89
Totale	***873***

TOSCANA	
Firenze	132
Siena	58
Massa Carrara	88
Pistoia	63
Lucca	96
Arezzo	27
Pisa	62
Livorno	40
Prato	33
Grosseto	31
Totale	***630***

CAMPANIA	
Napoli	171
Salerno	31
Caserta	41
Avellino	19
Benevento	3
In aggiornamento	7
Totale	***272***

LAZIO	
Roma	288
Frosinone	28
Viterbo	13
Rieti	3
Latina	20
in fase di aggiornamento	5
Totale	***357***

LIGURIA	
Savona	87
Imperia	64
Genova	231
La Spezia	52
in fase di aggiornamento	29
Totale	***463***

FRIULI VENEZIA GIULIA	
Trieste	148
Gorizia	20
Udine	88
Pordenone	45
Friuli in aggiornamento	
Totale	***301***

SICILIA	
Palermo	26
Agrigento	17
Caltanisetta	2
Catania	49
Enna	1
In aggiornamento	41
Messina	9
Ragusa	2
Siracusa	5
Trapani	4
Totale	***156***

PUGLIA	
BARI	42
BAT	17
BRINDISI	23
FOGGIA	52
LECCE	24
TARANTO	8
TOTALE	***166***

UMBRIA	
Perugia	47
Terni	29
Da aggiornare	31
Totale	***107***

ABRUZZO	
Teramo	9
Pescara	65
L'aquila	13
Chieti	25
Totale	***112***

MOLISE	
Campobasso	17
Totale	***17***

TRENTINO ALTO ADIGE	
Bolzano	173
Trento	206
Totale	***379***

SARDEGNA	
Città metropolitana di Cagliari	16
Sud Sardegna	5
Oristano	2
Nuoro	18
Sassari	6
Totale	***47***

BASILICATA	
Potenza	7
Matera	3
Totale	***10***

VALLE D'AOSTA	
AOSTA	42
Totale	***42***

CALABRIA	
Cosenza	15
Reggio Calabria	18
Catanzaro	5
Vibo Valentia	17
Crotone	5
Altro/In fase di aggiornamento	
Totale	***60***

**Totale Generale**	**21157**
