# Supplementary material for: COVID-19 in Italy: Dataset of the Italian Civil Protection Department
Source: Data Brief. 2020 Apr 10;30:105526. doi: 10.1016/j.dib.2020.105526 (PMC7178485; doi:10.1016/j.dib.2020.105526)
Supplement: Supplementary file 2 [file mmc2.zip › COVID-19/schede-riepilogative/province/dpc-covid19-ita-scheda-province-20200315.pdf]

Covid 19 - Ripartizione dei contagiati per provincia al 15/03/2020 ore 17

LOMBARDIA	
Bergamo	3416
Brescia	2473
Como	184
Cremona	1792
Lecco	344
Lodi	1320
Mantova	339
Milano	1750
Monza Brianza	327
Pavia	722
Sondrio	45
Varese	184
in fase di verifica e aggiornamento	376
Totale	***13272***

EMILIA-ROMAGNA	
Piacenza	1012
Parma	662
Reggio Emilia	185
Modena	367
Bologna	230
Ferrara	34
Ravenna	100
Forlì Cesena	78
Rimini	425
altro/in fase di verifica	
Totale	***3093***

VENETO	
PADOVA	658
VENEZIA	328
VICENZA	235
VERONA	335
BELLUNO	82
ROVIGO	27
TREVISO	413
altro/in fase di verifica	94
Totale	***2172***

MARCHE	
ANCONA	267
PESARO	712
MACERATA	94
FERMO	36
ASCOLI PICENO	14
altro/in fase di verifica	10
Totale	***1133***

PIEMONTE	
ALESSANDRIA	207
ASTI	87
BIELLA	50
CUNEO	61
Novara	71
Torino	359
VERCELLI	84
Verbano-Cusio-Ossola	50
altro/in fase di aggiornamento	142
Totale	***1111***

TOSCANA	
Firenze	162
Pistoia	79
Lucca	130
Siena	60
Massa Carrara	108
Arezzo	41
Pisa	71
Livorno	50
Grosseto	38
Prato	42
Totale	***781***

CAMPANIA	
NAPOLI	188
Salerno	49
Caserta	45
Avellino	37
Benevento	4
altro/in fase di verifica	10
Totale	***333***

LAZIO	
Roma	354
Frosinone	34
Viterbo	15
Rieti	4
Latina	23
in fase di aggiornamento	6
Totale	***436***

LIGURIA	
SAVONA	96
LA SPEZIA	60
IMPERIA	78
GENOVA	274
altro/in fase di verifica	51
Totale	***559***

FRIULI VENEZIA GIULIA	
Trieste	140
Gorizia	31
Udine	129
Pordenone	42
Friuli in aggiornamento	5
Totale	***347***

SICILIA	
AGRIGENTO	20
CALTANISSETTA	2
CATANIA	91
ENNA	2
MESSINA	10
PALERMO	33
RAGUSA	4
SIRACUSA	15
TRAPANI	11
Totale	***188***

PUGLIA	
BARI	58
BAT	22
BRINDISI	40
FOGGIA	62
LECCE	38
TARANTO	10
TOTALE	***230***

UMBRIA	
Perugia	86
Terni	57
Da aggiornare	
Totale	***143***

ABRUZZO	
L'Aquila	15
Chieti	30
Pescara	82
Teramo	10
Totale	***137***

MOLISE	
Campobasso	17
Totale	***17***

TRENTINO ALTO ADIGE	
Bolzano	204
Trento	378
Totale	***582***

SARDEGNA	
Città metropolitana di Cagliari	18
Sud Sardegna	3
Oristano	2
Nuoro	19
Sassari	35
Totale	***77***

BASILICATA	
Potenza	7
Matera	4
Totale	***11***

VALLE D'AOSTA	
AOSTA	57
Totale	***57***

CALABRIA	
COSENZA	17
REGGIO CALABRIA	22
CATANZARO	6
VIBO VALENTIA	6
CROTONE	17
Altro/In fase di aggiornamento	
Totale	***68***

**Totale Generale**	**24747**
