# Supplementary material for: COVID-19 in Italy: Dataset of the Italian Civil Protection Department
Source: Data Brief. 2020 Apr 10;30:105526. doi: 10.1016/j.dib.2020.105526 (PMC7178485; doi:10.1016/j.dib.2020.105526)
Supplement: Supplementary file 2 [file mmc2.zip › COVID-19/schede-riepilogative/province/dpc-covid19-ita-scheda-province-20200316.pdf]

Covid 19 - Ripartizione dei contagiati per provincia al 16/03/2020 ore 17

LOMBARDIA	
Bergamo	3760
Brescia	2918
Como	220
Cremona	1881
Lecco	386
Lodi	1362
Monza Brianza	346
Milano	1983
Mantova	382
Pavia	801
Sondrio	46
Varese	202
in fase di verifica e aggiornamento	362
Totale	***14649***

EMILIA-ROMAGNA	
Piacenza	1073
Parma	707
Reggio Emilia	241
Modena	440
Bologna	291
Ferrara	44
Ravenna	114
Forlì Cesena	103
Rimini	509
altro/in fase di verifica	
Totale	***3522***

VENETO	
PADOVA	715
ROVIGO	27
VENEZIA	356
VICENZA	287
VERONA	425
BELLUNO	101
TREVISO	452
altro/in fase di verifica	110
Totale	***2473***

MARCHE	
ANCONA	323
PESARO	733
MACERATA	117
FERMO	36
ASCOLI PICENO	21
altro/in fase di verifica	12
Totale	***1242***

PIEMONTE	
ALESSANDRIA	273
ASTI	87
BIELLA	67
CUNEO	91
Novara	109
Torino	542
VERCELLI	99
Verbano-Cusio-Ossola	58
altro/in fase di aggiornamento	190
Totale	***1516***

TOSCANA	
Firenze	186
Pistoia	90
Lucca	138
Siena	66
Massa Carrara	117
Arezzo	43
Pisa	78
Livorno	53
Grosseto	50
Prato	45
Totale	***866***

CAMPANIA	
NAPOLI	219
Salerno	57
Caserta	60
Avellino	49
Benevento	4
altro/in fase di verifica	11
Totale	***400***

LAZIO	
Roma	412
Frosinone	44
Viterbo	27
Rieti	11
Latina	23
in fase di aggiornamento	6
Totale	***523***

LIGURIA	
SAVONA	96
LA SPEZIA	60
IMPERIA	78
GENOVA	274
altro/in fase di verifica	159
Totale	***667***

FRIULI VENEZIA GIULIA	
Trieste	164
Gorizia	16
Udine	152
Pordenone	54
Friuli in aggiornamento	
Totale	***386***

SICILIA	
AGRIGENTO	22
CALTANISSETTA	4
CATANIA	96
ENNA	5
MESSINA	11
PALERMO	37
RAGUSA	4
SIRACUSA	21
TRAPANI	13
Totale	***213***

PUGLIA	
BARI	58
BAT	22
BRINDISI	40
FOGGIA	62
LECCE	38
TARANTO	10
TOTALE	***230***

UMBRIA	
Perugia	104
Terni	60
Da aggiornare	
Totale	***164***

ABRUZZO	
L'Aquila	18
Chieti	38
Pescara	101
Teramo	19
Totale	***176***

MOLISE	
Campobasso	21
Totale	***21***

TRENTINO ALTO ADIGE	
Bolzano	241
Trento	378
Totale	***619***

SARDEGNA	
Città metropolitana di Cagiari	24
Sud Sardegna	5
Oristano	2
Nuoro	19
Sassari	57
Totale	***107***

BASILICATA	
Potenza	8
Matera	4
Totale	***12***

VALLE D'AOSTA	
AOSTA	105
Totale	***105***

CALABRIA	
COSENZA	17
REGGIO CALABRIA	22
CATANZARO	6
VIBO VALENTIA	6
CROTONE	17
Altro/In fase di aggiornamento	21
Totale	***89***

**Totale Generale**	**27980**
