# Supplementary material for: COVID-19 in Italy: Dataset of the Italian Civil Protection Department
Source: Data Brief. 2020 Apr 10;30:105526. doi: 10.1016/j.dib.2020.105526 (PMC7178485; doi:10.1016/j.dib.2020.105526)
Supplement: Supplementary file 2 [file mmc2.zip › COVID-19/schede-riepilogative/province/dpc-covid19-ita-scheda-province-20200317.pdf]

Covid 19 - Ripartizione dei contagiati per provincia al 17/03/2020 ore 17

LOMBARDIA	
Bergamo	3993
Brescia	3300
Como	256
Cremona	2073
Lecco	440
Lodi	1418
Monza Brianza	376
Milano	2326
Mantova	465
Pavia	884
Sondrio	74
Varese	234
in fase di verifica e aggiornamento	381
Totale	***16220***

EMILIA-ROMAGNA	
Piacenza	1204
Parma	800
Reggio Emilia	299
Modena	460
Bologna	333
Ferrara	58
Ravenna	124
Forlì Cesena	144
Rimini (aggiornamento mancante)	509
altro/in fase di verifica	
Totale	***3931***

VENETO	
PADOVA	781
VENEZIA	378
VICENZA	325
VERONA	481
ROVIGO	28
BELLUNO	109
TREVISO	502
altro/in fase di verifica	100
Totale	***2704***

MARCHE	
ANCONA	356
PESARO	812
MACERATA	128
FERMO	36
ASCOLI PICENO	23
altro/in fase di verifica	16
Totale	***1371***

PIEMONTE	
ALESSANDRIA	323
ASTI	90
BIELLA	96
CUNEO	119
Novara	150
Torino	749
VERCELLI	113
Verbano-Cusio-Ossola	76
altro/in fase di aggiornamento	181
Totale	***1897***

TOSCANA	
Firenze	214
Pistoia	100
Lucca	179
Siena	73
Massa Carrara	143
Arezzo	71
Pisa	101
Livorno	59
Grosseto	60
Prato	53
Totale	***1053***

CAMPANIA	
Napoli	258
Salerno	65
Caserta	65
Avellino	56
Benevento	4
altro/in fase di verifica	12
Totale	***460***

LAZIO	
Roma	486
Frosinone	49
Rieti	12
Viterbo	31
Latina	23
altro/in fase di verifica	6
Totale	***607***

LIGURIA	
SAVONA	100
LA SPEZIA	65
IMPERIA	76
GENOVA	330
altro/in fase di verifica	207
Totale	***778***

FRIULI VENEZIA GIULIA	
Trieste	177
Gorizia	18
Udine	145
Pordenone	54
Friuli in aggiornamento	
Totale	***394***

SICILIA	
AGRIGENTO	22
CALTANISSETTA	4
CATANIA	108
ENNA	8
MESSINA	16
PALERMO	40
RAGUSA	4
SIRACUSA	21
TRAPANI	14
Totale	***237***

PUGLIA	
BARI	95
BAT	23
BRINDISI	52
FOGGIA	91
LECCE	62
TARANTO	17
TOTALE	***340***

UMBRIA	
Perugia	129
Terni	63
altro/in fase di aggiornamento	5
Totale	***197***

ABRUZZO	
L'Aquila	19
Chieti	41
Pescara	138
Teramo	31
Totale	***229***

MOLISE	
Campobasso	25
Totale	***25***

TRENTINO ALTO ADIGE	
Bolzano	291
Trento	385
Totale	***676***

SARDEGNA	
Città metropolitana di Cagliari	27
Sud Sardegna	5
Oristano	2
Nuoro	20
Sassari	63
Totale	***117***

BASILICATA	
Potenza	16
Matera	4
Totale	***20***

VALLE D'AOSTA	
AOSTA	136
Totale	***136***

CALABRIA	
COSENZA	27
REGGIO CALABRIA	46
CATANZARO	14
VIBO VALENTIA	6
CROTONE	21
Altro/In fase di aggiornamento	
Totale	***114***

**Totale Generale**	**31506**
