# Supplementary material for: COVID-19 in Italy: Dataset of the Italian Civil Protection Department
Source: Data Brief. 2020 Apr 10;30:105526. doi: 10.1016/j.dib.2020.105526 (PMC7178485; doi:10.1016/j.dib.2020.105526)
Supplement: Supplementary file 2 [file mmc2.zip › COVID-19/schede-riepilogative/province/dpc-covid19-ita-scheda-province-20200318.pdf]

Covid 19 - Ripartizione dei contagiati per provincia al 18/03/2020 ore 17

LOMBARDIA	
Bergamo	4305
Brescia	3784
Como	286
Cremona	2167
Lecco	466
Lodi	1445
Monza Brianza	401
Milano	2644
Mantova	514
Pavia	978
Sondrio	75
Varese	265
in fase di verifica e aggiornamento	383
Totale	***17713***

EMILIA-ROMAGNA	
Piacenza	1340
Parma (aggiornamento mancante)	800
Reggio Emilia	414
Modena	575
Bologna	395
Ferrara	64
Ravenna	153
Forlì Cesena	171
Rimini	613
altro/in fase di verifica	
Totale	***4525***

VENETO	
PADOVA	882
VENEZIA	426
ROVIGO	33
VICENZA	393
VERONA	626
BELLUNO	140
TREVISO	591
altro/in fase di verifica	123
Totale	***3214***

MARCHE	
ANCONA	403
PESARO	910
MACERATA	160
FERMO	46
ASCOLI PICENO	25
altro/in fase di verifica	24
Totale	***1568***

PIEMONTE	
ALESSANDRIA	374
ASTI	101
BIELLA	109
CUNEO	149
Novara	190
Torino	1042
VERCELLI	131
Verbano-Cusio-Ossola	89
altro/in fase di aggiornamento	156
Totale	***2341***

TOSCANA	
Firenze	295
Pistoia	122
Lucca	205
Siena	77
Massa Carrara	176
Arezzo	142
Pisa	124
Livorno	62
Grosseto	68
Prato	59
Totale	***1330***

CAMPANIA	
Napoli	258
Salerno	65
Caserta	65
Avellino	56
Benevento	4
altro/in fase di verifica	12
Totale	***460***

LAZIO	
Roma	590
Frosinone	54
Rieti	12
Viterbo	39
Latina	23
altro/in fase di verifica	6
Totale	***724***

LIGURIA	
SAVONA	126
LA SPEZIA	83
IMPERIA	101
GENOVA	378
altro/in fase di verifica	199
Totale	***887***

FRIULI VENEZIA GIULIA	
Trieste	180
Gorizia	25
Udine	181
Pordenone	76
Friuli in aggiornamento	
Totale	***462***

SICILIA	
AGRIGENTO	24
CALTANISSETTA	6
CATANIA	131
ENNA	8
MESSINA	16
PALERMO	47
RAGUSA	6
SIRACUSA	28
TRAPANI	16
Totale	***282***

PUGLIA	
BARI	112
BAT	23
BRINDISI	65
FOGGIA	91
LECCE	65
TARANTO	19
altro/in fase di aggiornamento	8
TOTALE	***383***

UMBRIA	
Perugia	164
Terni	74
altro/in fase di aggiornamento	9
Totale	***247***

ABRUZZO	
L'Aquila	20
Chieti	44
Pescara	160
Teramo	39
Totale	***263***

MOLISE	
Campobasso	24
Isernia	1
altro/in fase di aggiornamento	3
Totale	***28***

TRENTINO ALTO ADIGE	
Bolzano	376

Trento	455
Totale	***831***
**SARDEGNA**	
Città metropolitana di Cagliari	32
Sud Sardegna	5
Oristano	3
Nuoro	20
Sassari	74
Totale	***134***
**BASILICATA**	
Potenza	22
Matera	5
Totale	***27***
**VALLE D'AOSTA**	
AOSTA	165
Totale	***165***
**CALABRIA**	
COSENZA	31
REGGIO CALABRIA	47
CATANZARO	20
VIBO VALENTIA	8
CROTONE	23
Altro/In fase di aggiornamento	
Totale	***129***
**Totale Generale**	**35713**
