# Supplementary material for: COVID-19 in Italy: Dataset of the Italian Civil Protection Department
Source: Data Brief. 2020 Apr 10;30:105526. doi: 10.1016/j.dib.2020.105526 (PMC7178485; doi:10.1016/j.dib.2020.105526)
Supplement: Supplementary file 2 [file mmc2.zip › COVID-19/schede-riepilogative/regioni/dpc-covid19-ita-scheda-regioni-20200302.pdf]

Regione	AGGIORNAMENTO DEL 02/03/2020 ORE 18.00							
	POSITIVI AL nCoV				DIMESSI GUARITI	DECEDUTI	CASI TOTALI	TAMPONI
	ricoverati con sintomi	Terapia intensiva	Isolamento domiciliare	Totale attualmente positivi				
Lombardia	478	127	472	1077	139	38	1254	7925
Emilia Romagna	148	16	160	324		11	335	1973
Veneto	53	14	204	271		2	273	9782
Piemonte	12	2	37	51			51	434
Marche	17	6	11	34		1	35	137
Liguria	12	1	5	18	4		22	121
Campania	4		13	17			17	373
Toscana	7		5	12	1		13	613
Friuli V.G.			9	9			9	269
Sicilia	2		3	5	2		7	307
Lazio	3		1	4	3		7	773
Abruzzo	3		2	5			5	52
Puglia	2		2	4			4	278
Umbria			2	2			2	35
Bolzano	1			1			1	20
Calabria			1	1			1	39
Sardegna				0			0	29
Valle d'Aosta				0			0	11
Trento				0			0	122
Molise				0			0	13
Basilicata				0			0	39
TOTALE	742	166	927	1835	149	52	2036	23345

ATTUALMENTE POSITIVI	1835
TOTALE GUARITI	149
TOTALE DECEDUTI	52
CASI TOTALI	2036
