# Supplementary material for: COVID-19 in Italy: Dataset of the Italian Civil Protection Department
Source: Data Brief. 2020 Apr 10;30:105526. doi: 10.1016/j.dib.2020.105526 (PMC7178485; doi:10.1016/j.dib.2020.105526)
Supplement: Supplementary file 2 [file mmc2.zip › COVID-19/schede-riepilogative/regioni/dpc-covid19-ita-scheda-regioni-20200303.pdf]

PCM-DPC dati forniti dal Ministero della Salute

Regione	AGGIORNAMENTO DEL 03/03/2020 ORE 17.00							
	POSITIVI AL nCoV				DIMESSI GUARITI	DECEDUTI	CASI TOTALI	TAMPONI
	Ricoverati con sintomi	Terapia intensiva	Isolamento domiciliare	Totale attualmente positivi				
Lombardia	698	167	461	1326	139	55	1520	9577
Emilia Romagna	187	24	187	398	4	18	420	2012
Veneto	49	19	229	297	7	3	307	10176
Piemonte	13	3	40	56			56	458
Marche	27	13	19	59		2	61	200
Campania	11		19	30			30	405
Liguria	12	2	5	19	4	1	24	121
Toscana	10		8	18	1		19	697
Lazio	10		1	11	3		14	877
Friuli V.G.	1		12	13			13	354
Sicilia	2		3	5	2		7	307
Puglia	2		4	6			6	298
Abruzzo	5		1	6			6	52
Trento	1		3	4			4	122
Molise	3			3			3	13
Umbria	1	1	6	8			8	45
Bolzano	1			1			1	20
Calabria			1	1			1	39
Sardegna	1			1			1	29
Basilicata			1	1			1	42
Valle d'Aosta				0			0	12
TOTALE	1034	229	1000	2263	160	79	2502	25856

ATTUALMENTE POSITIVI	2263
TOTALE GUARITI	160
TOTALE DECEDUTI	79
CASI TOTALI	2502
