# Supplementary material for: COVID-19 in Italy: Dataset of the Italian Civil Protection Department
Source: Data Brief. 2020 Apr 10;30:105526. doi: 10.1016/j.dib.2020.105526 (PMC7178485; doi:10.1016/j.dib.2020.105526)
Supplement: Supplementary file 2 [file mmc2.zip › COVID-19/schede-riepilogative/regioni/dpc-covid19-ita-scheda-regioni-20200304.pdf]

PCM-DPC dati forniti dal Ministero della Salute

Regione	AGGIORNAMENTO DEL 04/03/2020 ORE 17.00							
	POSITIVI AL nCoV				DIMESSI GUARITI	DECEDUTI	CASI TOTALI	TAMPONI
	Ricoverati con sintomi	Terapia intensiva	Isolamento domiciliare	Totale attualmente positivi				
Lombardia	877	209	411	1497	250	73	1820	12138
Emilia Romagna	256	26	234	516	6	22	544	2500
Veneto	76	23	246	345	9	6	360	10515
Piemonte	26	13	43	82			82	543
Marche	34	15	31	80		4	84	288
Campania	11		20	31			31	429
Liguria	10	3	8	21	4	1	26	133
Toscana	15	2	20	37	1		38	776
Lazio	15	3	9	27	3		30	995
Friuli V.G.	3		15	18			18	376
Sicilia	5		11	16	2		18	367
Puglia	4		3	7	1	1	9	322
Abruzzo	7			7			7	85
Trento	1		4	5			5	122
Molise	3			3			3	19
Umbria	1	1	7	9			9	58
Bolzano	1			1			1	20
Calabria			1	1			1	46
Sardegna	1		1	2			2	42
Basilicata			1	1			1	48
Valle d'Aosta				0			0	15
TOTALE	1346	295	1065	2706	276	107	3089	29837

ATTUALMENTE POSITIVI	2706
TOTALE GUARITI	276
TOTALE DECEDUTI	107
CASI TOTALI	3089
