# Supplementary material for: COVID-19 in Italy: Dataset of the Italian Civil Protection Department
Source: Data Brief. 2020 Apr 10;30:105526. doi: 10.1016/j.dib.2020.105526 (PMC7178485; doi:10.1016/j.dib.2020.105526)
Supplement: Supplementary file 2 [file mmc2.zip › COVID-19/schede-riepilogative/regioni/dpc-covid19-ita-scheda-regioni-20200305.pdf]

PCM-DPC dati forniti dal Ministero della Salute

Regione	AGGIORNAMENTO DEL 05/03/2020 ORE 17.00							
	POSITIVI AL nCoV				DIMESSI GUARITI	DECEDUTI	CASI TOTALI	TAMPONI
	Ricoverati con sintomi	Terapia intensiva	Isolamento domiciliare	Totale attualmente positivi				
Lombardia	1169	244	364	1777	376	98	2251	12354
Emilia Romagna	327	32	299	658	10	30	698	2884
Veneto	92	24	264	380	17	10	407	11949
Marche	57	19	44	120		4	124	413
Piemonte	43	17	46	106		2	108	543
Toscana	26	3	31	60	1		61	776
Lazio	20	7	14	41	3		44	1175
Campania	12		33	45			45	471
Liguria	11	3	7	21	4	3	28	146
Friuli V.G.	4		17	21			21	397
Sicilia	5		11	16	2		18	367
Puglia	5	1	6	12	1	1	14	359
Umbria	1	1	7	9			9	88
Abruzzo	8			8			8	96
Trento	2		5	7			7	122
Molise	4		3	7			7	24
Valle d'Aosta			2	2			2	21
Calabria	1		1	2			2	53
Sardegna	2			2			2	50
Basilicata			1	1			1	54
Bolzano	1			1			1	20
TOTALE	1790	351	1155	3296	414	148	3858	32362

ATTUALMENTE POSITIVI	3296
TOTALE GUARITI	414
TOTALE DECEDUTI	148
CASI TOTALI	3858
