# Supplementary material for: COVID-19 in Italy: Dataset of the Italian Civil Protection Department
Source: Data Brief. 2020 Apr 10;30:105526. doi: 10.1016/j.dib.2020.105526 (PMC7178485; doi:10.1016/j.dib.2020.105526)
Supplement: Supplementary file 2 [file mmc2.zip › COVID-19/schede-riepilogative/regioni/dpc-covid19-ita-scheda-regioni-20200306.pdf]

PCM-DPC dati forniti dal Ministero della Salute

Regione	AGGIORNAMENTO DEL 06/03/2020 ORE 17.00							
	POSITIVI AL nCoV				DIMESSI GUARITI	DECEDUTI	CASI TOTALI	TAMPONI
	Ricoverati con sintomi	Terapia intensiva	Isolamento domiciliare	Totale attualmente positivi				
Lombardia	1622	309	77	2008	469	135	2612	13556
Emilia Romagna	397	53	366	816	17	37	870	3136
Veneto	117	27	310	454	22	12	488	13023
Marche	73	20	62	155		4	159	585
Piemonte	57	30	52	139		4	143	793
Toscana	35	5	38	78	1		79	1097
Lazio	26	8	16	50	3	1	54	1373
Campania	12		45	57			57	471
Liguria	12	5	7	24	5	3	32	229
Friuli V.G.	4		24	28	3		31	577
Sicilia	7		15	22	2		24	367
Puglia	5	1	9	15	1	1	17	395
Umbria	2	2	12	16			16	110
Molise	3	2	7	12			12	104
Trento	4		6	10			10	122
Abruzzo	9			9			9	96
Sardegna	2		3	5			5	99
Basilicata	1		2	3			3	63
Valle d'Aosta			7	7			7	28
Calabria	2		2	4			4	99
Bolzano	4			4			4	36
TOTALE	2394	462	1060	3916	523	197	4636	36359

ATTUALMENTE POSITIVI	3916
TOTALE GUARITI	523
TOTALE DECEDUTI	197
CASI TOTALI	4636
