# Supplementary material for: COVID-19 in Italy: Dataset of the Italian Civil Protection Department
Source: Data Brief. 2020 Apr 10;30:105526. doi: 10.1016/j.dib.2020.105526 (PMC7178485; doi:10.1016/j.dib.2020.105526)
Supplement: Supplementary file 2 [file mmc2.zip › COVID-19/schede-riepilogative/regioni/dpc-covid19-ita-scheda-regioni-20200307.pdf]

PCM-DPC dati forniti dal Ministero della Salute

Regione	AGGIORNAMENTO DEL 07/03/2020 ORE 17.00							
	POSITIVI AL nCoV				DIMESSI GUARITI	DECEDUTI	CASI TOTALI	TAMPONI
	Ricoverati con sintomi	Terapia intensiva	Isolamento domiciliare	Totale attualmente positivi				
Lombardia	1661	359	722	2742	524	154	3420	15778
Emilia Romagna	464	64	409	937	25	48	1010	3604
Veneto	123	41	341	505	25	13	543	14429
Piemonte	110	38	54	202		5	207	1046
Marche	94	36	71	201		6	207	816
Toscana	54	7	51	112	1		113	1331
Lazio	43	8	21	72	3	1	76	1582
Campania	16		45	61			61	612
Liguria	26	6	10	42	5	4	51	331
Friuli V.G.	7	1	31	39	3		42	577
Sicilia	8		25	33	2		35	643
Puglia	9	2	12	23	1	2	26	395
Umbria	2	2	20	24			24	134
Molise	3	2	9	14			14	112
Trento	6	1	7	14			14	194
Abruzzo	11			11			11	123
Bolzano	8		1	9			9	36
Valle d'Aosta	1		7	8			8	32
Sardegna	2		3	5			5	99
Calabria	2		2	4			4	113
Basilicata	1		2	3			3	75
TOTALE	2651	567	1843	5061	589	233	5883	42062

ATTUALMENTE POSITIVI	5061
TOTALE GUARITI	589
TOTALE DECEDUTI	233
CASI TOTALI	5883
