# Supplementary material for: COVID-19 in Italy: Dataset of the Italian Civil Protection Department
Source: Data Brief. 2020 Apr 10;30:105526. doi: 10.1016/j.dib.2020.105526 (PMC7178485; doi:10.1016/j.dib.2020.105526)
Supplement: Supplementary file 2 [file mmc2.zip › COVID-19/schede-riepilogative/regioni/dpc-covid19-ita-scheda-regioni-20200308.pdf]

PCM-DPC dati forniti dal Ministero della Salute

Regione	AGGIORNAMENTO 08/03/2020 ORE 17.00							
	POSITIVI AL nCoV				DIMESSI GUARITI	DECEDUTI	CASI TOTALI	TAMPONI
	Ricoverati con sintomi	Terapia intensiva	Isolamento domiciliare	Totale attualmente positivi				
Lombardia	2217	399	756	3372	550	267	4189	18534
Emilia Romagna	542	75	480	1097	27	56	1180	4344
Veneto	146	47	430	623	29	18	670	15918
Piemonte	245	45	65	355		5	360	1636
Marche	110	41	114	265		7	272	1025
Toscana	91	7	67	165	1		166	1618
Lazio	47	8	26	81	3	3	87	1929
Campania	30	7	63	100	1		101	980
Liguria	39	11	17	67	5	6	78	401
Friuli V.G.	7	1	45	53	3	1	57	997
Sicilia	18		33	51	2		53	791
Puglia	17	3	16	36	1	3	40	627
Umbria	2	2	22	26			26	168
Molise	4	2	8	14			14	116
Trento	7	2	14	23			23	228
Abruzzo	14		3	17			17	163
Bolzano	8		1	9			9	36
Valle d'Aosta	1		8	9			9	41
Sardegna	5		6	11			11	149
Calabria	5		4	9			9	113
Basilicata	2		2	4			4	123
TOTALE	3557	650	2180	6387	622	366	7375	49937

ATTUALMENTE POSITIVI	6387
TOTALE GUARITI	622
TOTALE DECEDUTI	366
CASI TOTALI	7375
