# Supplementary material for: COVID-19 in Italy: Dataset of the Italian Civil Protection Department
Source: Data Brief. 2020 Apr 10;30:105526. doi: 10.1016/j.dib.2020.105526 (PMC7178485; doi:10.1016/j.dib.2020.105526)
Supplement: Supplementary file 2 [file mmc2.zip › COVID-19/schede-riepilogative/regioni/dpc-covid19-ita-scheda-regioni-20200309.pdf]

PCM-DPC dati forniti dal Ministero della Salute

AGGIORNAMENTO 09/03/2020 ORE 17.00

Regione	POSITIVI AL nCoV				DIMESSI GUARITI	DECEDUTI	CASI TOTALI	TAMPONI
	Ricoverati con sintomi	Terapia intensiva	Isolamento domiciliare	Totale attualmente positivi				
Lombardia	2802	440	1248	4490	646	333	5469	20135
Emilia Romagna	576	90	620	1286	30	70	1386	4906
Veneto	186	51	457	694	30	20	744	15956
Piemonte	222	50	65	337		13	350	1681
Marche	136	47	130	313		10	323	1250
Toscana	107	9	90	206	1	1	208	2018
Lazio	55	8	31	94	3	5	102	1929
Campania	42	8	69	119	1		120	980
Liguria	60	17	20	97	5	7	109	611
Friuli V.G.	18	1	70	89	3	1	93	1344
Sicilia	19		33	52	2		54	836
Puglia	20	6	20	46	1	3	50	685
Trento	10	2	21	33			33	267
Abruzzo	25		5	30			30	237
Umbria	4	2	22	28			28	183
Molise	4	2	8	14			14	212
Sardegna	8		11	19			19	185
Valle d'Aosta	4		11	15			15	67
Calabria	8		1	9	2		11	173
Bolzano	8		1	9			9	36
Basilicata	2		3	5			5	135
TOTALE	4316	733	2936	7985	724	463	9172	53826

ATTUALMENTE POSITIVI	7985
TOTALE GUARITI	724
TOTALE DECEDUTI	463
CASI TOTALI	9172
