# Supplementary material for: COVID-19 in Italy: Dataset of the Italian Civil Protection Department
Source: Data Brief. 2020 Apr 10;30:105526. doi: 10.1016/j.dib.2020.105526 (PMC7178485; doi:10.1016/j.dib.2020.105526)
Supplement: Supplementary file 2 [file mmc2.zip › COVID-19/schede-riepilogative/regioni/dpc-covid19-ita-scheda-regioni-20200310.pdf]

PCM-DPC dati forniti dal Ministero della Salute

Regione	AGGIORNAMENTO 10/03/2020 ORE 17.00							
	POSITIVI AL nCoV				DIMESSI GUARITI	DECEDUTI	CASI TOTALI	TAMPONI
	Ricoverati con sintomi	Terapia intensiva	Isolamento domiciliare	Totale attualmente positivi				
Lombardia	3319	466	642	**4427**	**896**	**468**	**5791**	**21479**
Emilia Romagna	669	98	650	**1417**	**31**	**85**	**1533**	**5494**
Veneto	204	67	512	**783**	**47**	**26**	**856**	**16643**
Piemonte	306	66	64	**436**		**17**	**453**	**2374**
Marche	152	54	175	**381**		**13**	**394**	**1437**
Toscana	91	40	129	**260**	**3**	**1**	**264**	**2573**
Lazio	50	15	34	**99**	**11**	**6**	**116**	**3591**
Campania	33	8	85	**126**	**1**		**127**	**1141**
Liguria	57	29	42	**128**	**5**	**8**	**141**	**694**
Friuli V.G.	27	6	77	**110**	**3**	**3**	**116**	**1602**
Sicilia	17	2	41	**60**	**2**		**62**	**955**
Puglia	28	6	21	**55**	**1**	**3**	**59**	**747**
Trento	19	3	28	**50**	2		**52**	**399**
Abruzzo	17	9	11	**37**		**1**	**38**	**310**
Umbria	8	2	27	**37**			**37**	**260**
Molise	3	3	9	**15**			**15**	**225**
Sardegna	9		11	**20**			**20**	**204**
Valle d'Aosta	2		15	**17**			**17**	**89**
Calabria	8	2	1	**11**	**2**		**13**	**360**
Bolzano	17	1	20	**38**			**38**	**36**
Basilicata	2		5	**7**			**7**	**148**
**TOTALE**	**5038**	**877**	**2599**	**8514**	**1004**	**631**	**10149**	**60761**

**ATTUALMENTE POSITIVI**	**8514**
**TOTALE GUARITI**	**1004**
**TOTALE DECEDUTI**	**631**
**CASI TOTALI**	**10149**
