# Supplementary material for: COVID-19 in Italy: Dataset of the Italian Civil Protection Department
Source: Data Brief. 2020 Apr 10;30:105526. doi: 10.1016/j.dib.2020.105526 (PMC7178485; doi:10.1016/j.dib.2020.105526)
Supplement: Supplementary file 2 [file mmc2.zip › COVID-19/schede-riepilogative/regioni/dpc-covid19-ita-scheda-regioni-20200311.pdf]

PCM-DPC dati forniti dal Ministero della Salute

Regione	AGGIORNAMENTO 11/03/2020 ORE 17.00							
	POSITIVI AL nCoV				DIMESSI GUARITI	DECEDUTI	CASI TOTALI	TAMPONI
	Ricoverati con sintomi	Terapia intensiva	Isolamento domiciliare	Totale attualmente positivi				
Lombardia	3852	560	1351	5763	900	617	7280	25629
Emilia Romagna	745	104	739	1588	38	113	1739	6640
Veneto	262	68	610	940	54	29	1023	21400
Piemonte	319	75	86	480		21	501	2431
Marche	212	66	183	461		18	479	1656
Toscana	87	54	173	314	5	1	320	2804
Liguria	74	34	73	181	5	8	194	1025
Campania	56	11	82	149	4	1	154	1375
Lazio	67	18	40	125	19	6	150	3591
Friuli V.G.	16	5	89	110	10	6	126	2073
Puglia	38	4	29	71	1	5	77	909
Trento	28	4	42	74	3		77	527
Bolzano	8	4	63	75			75	75
Sicilia	23	1	57	81	2		83	1194
Umbria	7	5	32	44	2		46	340
Abruzzo	17	9	11	37		1	38	310
Sardegna	10		27	37			37	283
Valle d'Aosta	2		17	19		1	20	99
Calabria	10	2	5	17	2		19	405
Molise	4	3	9	16			16	233
Basilicata	1	1	6	8			8	155
**TOTALE**	**5838**	**1028**	**3724**	**10590**	**1045**	**827**	**12462**	**73154**

ATTUALMENTE POSITIVI	10590
TOTALE GUARITI	1045
TOTALE DECEDUTI	827
CASI TOTALI	12462
