# Supplementary material for: COVID-19 in Italy: Dataset of the Italian Civil Protection Department
Source: Data Brief. 2020 Apr 10;30:105526. doi: 10.1016/j.dib.2020.105526 (PMC7178485; doi:10.1016/j.dib.2020.105526)
Supplement: Supplementary file 2 [file mmc2.zip › COVID-19/schede-riepilogative/regioni/dpc-covid19-ita-scheda-regioni-20200312.pdf]

PCM-DPC dati forniti dal Ministero della Salute

Regione	AGGIORNAMENTO 12/03/2020 ORE 17.00							
	POSITIVI AL nCoV				DIMESSI GUARITI	DECEDUTI	CASI TOTALI	TAMPONI
	Ricoverati con sintomi	Terapia intensiva	Isolamento domiciliare	Totale attualmente positivi				
Lombardia	4247	605	2044	6896	1085	744	8725	29534
Emilia Romagna	814	112	832	1758	43	146	1947	7600
Veneto	360	85	852	1297	55	32	1384	23438
Piemonte	368	97	89	554		26	580	2879
Marche	254	76	240	570		22	592	1907
Toscana	100	59	193	352	7	5	364	3165
Liguria	100	36	107	243	20	11	274	1174
Campania	56	11	107	174	4	1	179	1551
Lazio	85	20	67	172	19	9	200	5592
Friuli V.G.	23	10	115	148	11	8	167	2604
Puglia	58	2	38	98	1	5	104	1269
Trento	43	5	54	102	4	1	107	593
Bolzano	21	4	78	103		1	104	607
Sicilia	28	5	78	111	2	2	115	1477
Umbria	8	8	46	62	2		64	458
Abruzzo	47	12	19	78	4	2	84	867
Sardegna	12		27	39			39	302
Valle d'Aosta	7		19	26		1	27	118
Calabria	14	2	16	32	1		33	483
Molise	4	3	9	16			16	238
Basilicata	1	1	6	8			8	155
TOTALE	6650	1153	5036	12839	1258	1016	15113	86011

ATTUALMENTE POSITIVI	12839
TOTALE GUARITI	1258
TOTALE DECEDUTI	1016
CASI TOTALI	15113
