# Supplementary material for: COVID-19 in Italy: Dataset of the Italian Civil Protection Department
Source: Data Brief. 2020 Apr 10;30:105526. doi: 10.1016/j.dib.2020.105526 (PMC7178485; doi:10.1016/j.dib.2020.105526)
Supplement: Supplementary file 2 [file mmc2.zip › COVID-19/schede-riepilogative/regioni/dpc-covid19-ita-scheda-regioni-20200313.pdf]

PCM-DPC dati forniti dal Ministero della Salute

Regione	AGGIORNAMENTO 13/03/2020 ORE 17.00							
	POSITIVI AL nCoV				DIMESSI GUARITI	DECEDUTI	CASI TOTALI	TAMPONI
	Ricoverati con sintomi	Terapia intensiva	Isolamento domiciliare	Totale attualmente positivi				
Lombardia	4435	650	2647	7732	1198	890	9820	32700
Emilia Romagna	942	128	941	2011	51	201	2263	8787
Veneto	366	107	980	1453	100	42	1595	25691
Piemonte	556	135	103	794		46	840	3105
Marche	337	85	276	698		27	725	2218
Toscana	134	77	244	455	10	5	470	4049
Liguria	128	44	132	304	24	17	345	1442
Lazio	122	24	96	242	24	11	277	6491
Friuli V.G.	59	8	169	236	11	10	257	3149
Campania	60	19	134	213	5	2	220	1671
Puglia	77	2	42	121	3	5	129	1449
Bolzano	20	5	98	123		2	125	811
Sicilia	37	7	82	126	2	2	130	1950
Trento	58	6	93	157	4	2	163	846
Abruzzo	42	14	27	83	4	2	89	958
Umbria	11	10	52	73	2	1	76	576
Sardegna	12		31	43			43	504
Calabria	18	3	16	37	1		38	504
Valle d'Aosta	6		21	27		1	28	189
Molise	5	3	9	17			17	243
Basilicata	1	1	8	10			10	155
TOTALE	7426	1328	6201	14955	1439	1266	17660	97488

ATTUALMENTE POSITIVI	14955
TOTALE GUARITI	1439
TOTALE DECEDUTI	1266
CASI TOTALI	17660
