# Supplementary material for: COVID-19 in Italy: Dataset of the Italian Civil Protection Department
Source: Data Brief. 2020 Apr 10;30:105526. doi: 10.1016/j.dib.2020.105526 (PMC7178485; doi:10.1016/j.dib.2020.105526)
Supplement: Supplementary file 2 [file mmc2.zip › COVID-19/schede-riepilogative/regioni/dpc-covid19-ita-scheda-regioni-20200314.pdf]

PCM-DPC dati forniti dal Ministero della Salute

Regione	AGGIORNAMENTO 14/03/2020 ORE 17.00							
	POSITIVI AL nCoV				DIMESSI GUARITI	DECEDUTI	CASI TOTALI	TAMPONI
	Ricoverati con sintomi	Terapia intensiva	Isolamento domiciliare	Totale attualmente positivi				
Lombardia	4898	732	3429	9059	1660	966	11685	37138
Emilia Romagna	1076	152	1121	2349	54	241	2644	10043
Veneto	366	119	1290	1775	107	55	1937	26980
Marche	449	93	321	863		36	899	2561
Piemonte	538	150	126	814		59	873	3680
Toscana	160	87	367	614	10	6	630	4595
Liguria	213	62	109	384	52	27	463	1750
Lazio	181	25	114	320	24	13	357	7335
Campania	72	17	154	243	23	6	272	1936
Friuli V.G.	67	11	193	271	17	13	301	3376
Trento	68	12	119	199	5	2	206	1006
Bolzano	26	7	137	170		3	173	1135
Puglia	91	6	59	156	2	8	166	1681
Sicilia	42	11	97	150	4	2	156	2100
Umbria	21	11	71	103	3	1	107	748
Abruzzo	51	14	41	106	4	2	112	1232
Calabria	22	4	33	59	1		60	711
Sardegna	14		33	47			47	530
Valle d'Aosta	12		29	41		1	42	231
Molise	5	3	9	17			17	247
Basilicata		2	8	10			10	155
TOTALE	8372	1518	7860	17750	1966	1441	21157	109170

ATTUALMENTE POSITIVI	17750
TOTALE GUARITI	1966
TOTALE DECEDUTI	1441
CASI TOTALI	21157
