# Supplementary material for: COVID-19 in Italy: Dataset of the Italian Civil Protection Department
Source: Data Brief. 2020 Apr 10;30:105526. doi: 10.1016/j.dib.2020.105526 (PMC7178485; doi:10.1016/j.dib.2020.105526)
Supplement: Supplementary file 2 [file mmc2.zip › COVID-19/schede-riepilogative/regioni/dpc-covid19-ita-scheda-regioni-20200315.pdf]

Regione	AGGIORNAMENTO 15/03/2020 ORE 17.00							
	POSITIVI AL nCoV				DIMESSI/ GUARITI	DECEDUTI	CASI TOTALI	TAMPONI
	Ricoverati con sintomi	Terapia intensiva	Isolamento domiciliare	Totale attualmente positivi				
Lombardia	5500	767	3776	**10043**	**2011**	**1218**	**13272**	**40369**
Emilia Romagna	1215	169	1357	**2741**	**68**	**284**	**3093**	**12054**
Veneto	426	129	1434	**1989**	**120**	**63**	**2172**	**32546**
Marche	521	98	468	**1087**		**46**	**1133**	**2946**
Piemonte	726	171	133	**1030**		**81**	**1111**	**4375**
Toscana	175	107	481	**763**	**10**	**8**	**781**	**5132**
Liguria	253	66	174	**493**	**33**	**33**	**559**	**1973**
Lazio	223	31	142	**396**	**24**	**16**	**436**	**8345**
Campania	73	22	201	**296**	**28**	**9**	**333**	**2213**
Friuli V.G.	98	12	206	**316**	**17**	**14**	**347**	**3407**
Trento	73	19	275	**367**	**5**	**6**	**378**	**1006**
Bolzano	50	4	145	**199**		**5**	**204**	**1497**
Puglia	116	6	90	**212**	**2**	**16**	**230**	**2017**
Sicilia	56	15	108	**179**	**7**	**2**	**188**	**2452**
Umbria	25	13	101	**139**	**3**	**1**	**143**	**965**
Abruzzo	72	28	28	**128**	**6**	**3**	**137**	**1419**
Calabria	32	6	28	**66**	**1**	**1**	**68**	**884**
Sardegna	16		59	**75**		**2**	**77**	**613**
Valle d'Aosta	10	3	43	**56**		**1**	**57**	**230**
Molise	3	4	10	**17**			**17**	**248**
Basilicata		2	9	**11**			**11**	**208**
**TOTALE**	**9663**	**1672**	**9268**	**20603**	**2335**	**1809**	**24747**	**124899**

**ATTUALMENTE POSITIVI**	**20603**
**TOTALE GUARITI**	**2335**
**TOTALE DECEDUTI**	**1809**
**CASI TOTALI**	**24747**
