# Supplementary material for: COVID-19 in Italy: Dataset of the Italian Civil Protection Department
Source: Data Brief. 2020 Apr 10;30:105526. doi: 10.1016/j.dib.2020.105526 (PMC7178485; doi:10.1016/j.dib.2020.105526)
Supplement: Supplementary file 2 [file mmc2.zip › COVID-19/schede-riepilogative/regioni/dpc-covid19-ita-scheda-regioni-20200316.pdf]

PCM-DPC dati forniti dal Ministero della Salute

Regione	AGGIORNAMENTO 16/03/2020 ORE 17.00							
	POSITIVI AL nCoV				DIMESSI/ GUARITI	DECEDUTI	CASI TOTALI	TAMPONI
	Ricoverati con sintomi	Terapia intensiva	Isolamento domiciliare	Totale attualmente positivi				
Lombardia	6171	823	3867	10861	2368	1420	14.649	43.565
Emilia Romagna	1362	197	1529	3088	88	346	3.522	13.096
Veneto	498	156	1620	2274	130	69	2.473	35.052
Marche	528	110	547	1185		57	1.242	3.225
Piemonte	1045	186	174	1405		111	1.516	5.588
Toscana	175	107	559	841	11	14	866	5.910
Liguria	255	73	247	575	42	50	667	2.189
Lazio	267	31	174	472	32	19	523	9.330
Campania	103	22	238	363	28	9	400	2.517
Friuli V.G.	96	19	231	346	18	22	386	4.851
Trento	73	19	275	367	5	6	378	1.006
Bolzano	53	11	171	235		6	241	1.740
Puglia	116	6	90	212	2	16	230	2.017
Sicilia	75	20	108	203	8	2	213	2.653
Umbria	30	15	114	159	4	1	164	1.093
Abruzzo	71	37	57	165	7	4	176	1.533
Calabria	36	7	44	87	1	1	89	1.030
Sardegna	39		66	105		2	107	797
Valle d'Aosta	28	5	70	103		2	105	287
Molise	3	5	7	15	5	1	21	253
Basilicata	1	2	9	12			12	230
TOTALE	11.025	1.851	10.197	23.073	2.749	2.158	27.980	137.962

ATTUALMENTE POSITIVI	23073
TOTALE GUARITI	2749
TOTALE DECEDUTI	2158
CASI TOTALI	27980
