# Supplementary material for: COVID-19 in Italy: Dataset of the Italian Civil Protection Department
Source: Data Brief. 2020 Apr 10;30:105526. doi: 10.1016/j.dib.2020.105526 (PMC7178485; doi:10.1016/j.dib.2020.105526)
Supplement: Supplementary file 2 [file mmc2.zip › COVID-19/schede-riepilogative/regioni/dpc-covid19-ita-scheda-regioni-20200317.pdf]

PCM-DPC dati forniti dal Ministero della Salute

Regione	AGGIORNAMENTO 17/03/2020 ORE 17.00							
	POSITIVI AL nCoV				DIMESSI/ GUARITI	DECEDUTI	CASI TOTALI	TAMPONI
	Ricoverati con sintomi	Terapia intensiva	Isolamento domiciliare	Totale attualmente positivi				
Lombardia	6953	879	4263	12095	2485	1640	16.220	46.449
Emilia Romagna	1566	223	1615	3404	134	393	3.931	14.510
Veneto	548	171	1769	2488	136	80	2.704	35.478
Piemonte	1378	206	180	1764		133	1.897	6.543
Marche	599	109	594	1302		69	1.371	3.225
Toscana	329	143	552	1024	12	17	1.053	6.727
Liguria	299	85	277	661	57	60	778	2.509
Lazio	314	44	192	550	34	23	607	9.436
Campania	127	24	272	423	28	9	460	2.685
Friuli V.G.	104	21	222	347	17	30	394	4.958
Trento	107	22	239	368	10	7	385	1.727
Bolzano	71	11	200	282	1	8	291	2.149
Puglia	155	14	151	320	2	18	340	3.077
Sicilia	86	28	112	226	8	3	237	2.916
Abruzzo	94	32	90	216	7	6	229	1.688
Umbria	36	21	135	192	4	1	197	1.323
Valle d'Aosta	35	6	93	134		2	136	398
Sardegna	36	4	75	115		2	117	1.003
Calabria	45	10	57	112	1	1	114	1.293
Molise	7	5	7	19	5	1	25	301
Basilicata	5	2	13	20			20	262
TOTALE	12.894	2.060	11.108	26.062	2.941	2.503	31.506	148.657

ATTUALMENTE POSITIVI	26062
TOTALE GUARITI	2941
TOTALE DECEDUTI	2503
CASI TOTALI	31506
