# Supplementary material for: COVID-19 in Italy: Dataset of the Italian Civil Protection Department
Source: Data Brief. 2020 Apr 10;30:105526. doi: 10.1016/j.dib.2020.105526 (PMC7178485; doi:10.1016/j.dib.2020.105526)
Supplement: Supplementary file 2 [file mmc2.zip › COVID-19/schede-riepilogative/regioni/dpc-covid19-ita-scheda-regioni-20200318.pdf]

PCM-DPC dati forniti dal Ministero della Salute

Regione	AGGIORNAMENTO 18/03/2020 ORE 17.00							
	POSITIVI AL nCoV				DIMESSI/ GUARITI	DECEDUTI	CASI TOTALI	TAMPONI
	Ricoverati con sintomi	Terapia intensiva	Isolamento domiciliare	Totale attualmente positivi				
Lombardia	7285	924	4057	12266	3488	1959	17.713	48.983
Emilia Romagna	1784	247	1884	3915	152	458	4.525	15.461
Veneto	646	195	2112	2953	167	94	3.214	40.841
Piemonte	1780	227	180	2187		154	2.341	7.516
Marche	638	119	719	1476		92	1.568	4.109
Toscana	427	160	704	1291	17	22	1.330	7.606
Liguria	401	100	243	744	70	73	887	2.912
Lazio	374	44	232	650	42	32	724	11.145
Campania	127	24	272	423	28	9	460	2.685
Friuli V.G.	128	27	261	416	15	31	462	4.958
Trento	141	22	273	436	12	7	455	2.187
Bolzano	79	18	269	366	1	9	376	2.844
Puglia	156	30	176	362	2	19	383	3.433
Sicilia	100	29	138	267	12	3	282	3.294
Abruzzo	99	41	109	249	7	7	263	2.054
Umbria	54	21	166	241	4	2	247	1.601
Valle d'Aosta	41	3	118	162		3	165	486
Sardegna	42	7	83	132		2	134	1.135
Calabria	45	11	70	126	2	1	129	1.668
Molise	7	6	8	21	6	1	28	361
Basilicata	9	2	16	27			27	262
TOTALE	14.363	2.257	12.090	28.710	4.025	2.978	35.713	165.541

ATTUALMENTE POSITIVI	28710
TOTALE GUARITI	4025
TOTALE DECEDUTI	2978
CASI TOTALI	35713
